# Point-of-Care and Dual-Response Detection of Hydrazine/Hypochlorite-Based on a Smart Hydrogel Sensor and Applications in Information Security and Bioimaging

**DOI:** 10.3390/molecules28093896

**Published:** 2023-05-05

**Authors:** Man Du, Yue Zhang, Zhice Xu, Zhipeng Dong, Shuchun Zhao, Hongxia Du, Hua Zhao

**Affiliations:** 1 College of Chemical and Pharmaceutical Engineering, Hebei University of Science and Technology, Shijiazhuang 050018, China; 2 Hebei Lansheng Biotech Co., Ltd., Shijiazhuang 052260, China

**Keywords:** fluorescence probe, hydrazine, hypochlorite, hydrogel, information encryption, bioimaging

## Abstract

A novel dual-response fluorescence probe (**XBT-CN**) was developed by using a fluorescence priming strategy for quantitative monitoring and visualization of hydrazine (N_2_H_4_) and hypochlorite (ClO^−^). With the addition of N_2_H_4_/ClO^−^, the cleavage reaction of C=C bond initiated by N_2_H_4_/ClO^−^ was transformed into corresponding hydrazone and aldehyde derivatives, inducing the probe **XBT-CN** appeared a fluorescence “off-on” response, which was verified by DFT calculation. HRMS spectra were also conducted to confirm the sensitive mechanism of **XBT-CN** to N_2_H_4_ and ClO^−^. The probe **XBT-CN** had an obvious fluorescence response to N_2_H_4_ and ClO^−^, which caused a significant color change in unprotected eyes. In addition, the detection limits of **XBT-CN** for N_2_H_4_ and ClO^−^ were 27 nM and 34 nM, respectively. Interference tests showed that other competitive analytes could hardly interfere with the detection of N_2_H_4_ and ClO^−^ in a complex environment. In order to realize the point-of-care detection of N_2_H_4_ and ClO^−^, an **XBT-CN**@hydrogel test kit combined with a portable smartphone was developed. Furthermore, the portable test kit has been applied to the detection of N_2_H_4_ and ClO^−^ in a real-world environment and food samples, and a series of good results have been achieved. Attractively, we demonstrated that **XBT-CN**@hydrogel was successfully applied as an encryption ink in the field of information security. Finally, the probe can also be used to monitor and distinguish N_2_H_4_ and ClO^−^ in living cells, exhibiting excellent biocompatibility and low cytotoxicity.

## 1. Introduction

As a highly reactive alkali-reducing agent, hydrazine (N_2_H_4_) is extensively applied in chemical enterprises, agriculture, pharmaceuticals, missile, aerospace, and other various fields [1,2,3]. Although N_2_H_4_ is widely used, it seriously harms the health of human and can be absorbed by the body, causing serious damage to the organs and system, such as the kidneys, liver, lungs, central nervous system, and respiratory tract, even leading to death [4,5,6,7]. Moreover, due to its high carcinogenicity, N_2_H_4_ is included in the list of Class 2A carcinogens according to the preliminary list of carcinogens published by the World Health Organization (WHO) [8,9,10]. The low threshold limit (TLV) of N_2_H_4_ has been certified as 10 ppb by the US Environmental Protection Agency (EPA) [11,12]. Furthermore, hypochlorite (ClO^−^) is an important member of reactive oxygen species (ROS) and plays a significant role in regulating many biological activities [13,14,15]. In living organisms, HOCl is produced endogenously by myeloperoxidase (MPO) which catalases the oxidation of chloride ions in the presence of hydrogen peroxide [16]. In addition, as a strong oxidant, ClO^−^ has been widely applied among people’s daily lives due to its high oxidizability and antibacterial activity, such as household bleach, drinking water disinfection, sewage purification, cooling water treatment, and bleach in paper and textile industries [17,18,19]. However, excessive endogenous ClO^−^ can lead to a series of lesions and diseases, including tissue damage, cardiovascular disease, inflammatory disease, neuronal degeneration, lung injury, atherosclerosis, and cancer [20,21,22]. Therefore, for the sake of human health and safety, it is indispensable to develop point-of-care analytical methods to selectively and sensitively detect N_2_H_4_ and ClO^−^ in the environment and food.

Traditional determination techniques of N_2_H_4_ and ClO^−^ include titration, capillary electrophoresis, electrochemical analysis, chemiluminescence, and chromatography [23,24,25,26,27,28,29,30]. However, these conventional methods have many shortcomings, such as time-consuming, complex sample processing, and great destructiveness to biological samples to be tested. Compared with the traditional methods, fluorescent probes have the advantages of easy realization, high sensitivity, good selectivity, no need for sample preparation, and are more attractive [31,32]. Up to now, various fluorescent probes of N_2_H_4_ [33,34,35] and ClO^−^ [36,37,38,39,40] have been reported. In order to give a correct view of N_2_H_4_/ClO^−^ probes developed in recent three years, a brief comparison with some recently developed N_2_H_4_/ClO^−^ fluorescent chemical sensors is also listed in Table S1.

Although some of these fluorescent probes exhibit powerful detection ability for N_2_H_4_ or ClO^−^, few of them can identify N_2_H_4_ and ClO^−^ at the same time. However, N_2_H_4_ and ClO^−^ often exist in the same sample from the environment or food. If two different fluorescence probes are used for determination, it will cost a high quantity of human effort and resources to detect accurately. The dual-response fluorescence probe can detect two kinds of analytes with different fluorescence responses using only a single sensor, which can greatly improve testing efficiency and decrease product cost. The mechanisms of single sensors that can detect N_2_H_4_ and ClO^−^ simultaneously with two different outputs include two main kinds. One kind simultaneously introduces S=O and C=C bonds to the molecule of the fluorescence probe because N_2_H_4_ can induce C=C double bond cleavage and ClO^−^ can induced sulfur oxidation respectively. According to this, Li’s team synthesized a carbazole derivative probe for detecting N_2_H_4_ and ClO^−^ in 2020 [41]. Hu’s team designed a fluorescent sensor with two action sites for the separate detection of N_2_H_4_ and ClO^−^ in 2021 [42]. Xiao’s team also developed a colorimetric two-photon multianalyte sensor with a spirobifluorene motif for rapid detection of N_2_H_4_ and ClO^−^ in 2022 [43]. Further, another mechanism is that N_2_H_4_ and ClO^−^ could both trigger the same C=C bond cleavage generating two detecting products with different fluorescence responses. Based on this strategy, Shen’s team prepared a chemosensor detecting N_2_H_4_/ClO^−^ with different fluorescence responses by condensation of naphthoic aldehyde and phenobarbital acid in 2020 [44]. Mondal’s team designed a ratiometric fluorescent probe detecting N_2_H_4_ and ClO^−^ via a “dye-release” mechanism in 2022 [45]. There are only a few fluorescent probes mentioned above that can simultaneously detect N_2_H_4_ and ClO^−^. In summary, it is still imperative to develop a fluorescent probe that can recognize and distinguish N_2_H_4_ and ClO^−^ at the same time.

**Figure 1:**
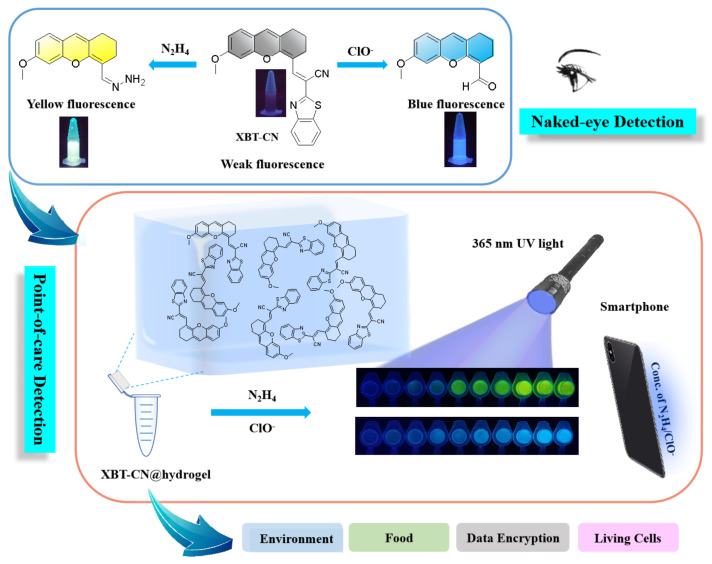
Illustration of the probe **XBT-CN** for detecting N_2_H_4_ and ClO^−^ and its practical application in real samples.

In view of the above results, we designed and synthesized a novel dual-response chemical probe (**XBT-CN**), which is composed of anthracene aldehyde and benzothiazole acetonitrile by a condensation reaction, and it has varying fluorescence responses to various analytes. In one case, **XBT-CN** can react with N_2_H_4_ to form a yellow hydrazone derivative via a nucleophilic reaction to the C=C bond, with the fluorescence changing from subtle to luminous yellow. On the other hand, the C=C bond of **XBT-CN** can be cleaved via ClO^−^ oxidation to generate an aldehyde derivative, following the fluorescence changes from subtle to brilliant blue, which shows high sensitivity and selectivity. Therefore, **XBT-CN** can detect N_2_H_4_ and ClO^−^ in fluorescence emission channels. Moreover, we developed a portable **XBT-CN**@hydrogel test kit integrated with a color analysis on smartphones for point-of-care detection of N_2_H_4_ and ClO^−^. Notably, the portable hydrogel kit was successfully applied to the quantitative detection of N_2_H_4_ and ClO^−^ in real samples from environment and food, providing a powerful tool for practical point-of-care detection in the environment and food safety. In addition, we also employ it as an encryption ink in the field of information security. Finally, **XBT-CN** has been practically applied to biological imaging of N_2_H_4_ and ClO^−^ in living cells (Figure 1).

## 2. Results and Discussion

### 2.1. Design and Synthesis of the Probe **XBT-CN**

**Scheme 1:**
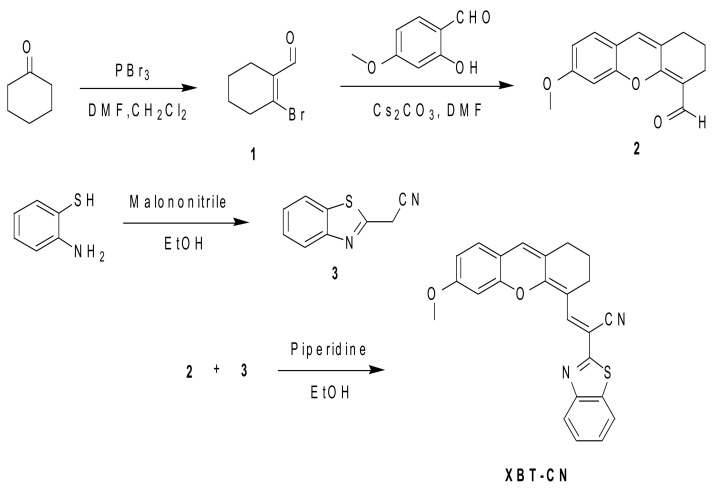
Synthesis of the probe **XBT-CN**.

**XBT-CN** probe was designed with xanthone as a fluorescent group and benzothiazole acetonitrile as a recognition unit. Xanthone is chosen in this paper because of its high emission efficiency and simple preparation process. Benzothiazole acetonitrile, as a typical recognition group of N_2_H_4_, is a good reaction site for recognizing N_2_H_4_ due to C=C bridging group and is introduced into xanthone derivative to detect N_2_H_4_ via a nucleophilic reaction to form a yellow hydrazone derivative. In addition, the strong electron withdrawing group of benzothiazole acetonitrile makes the C=C bond easier to be attacked by ClO^−^, which leads to the cancellation of the conjugated system and the formation of aldehyde conjugated product of the oxidative nucleophilic reaction. The design strategy of **XBT-CN** reacts with N_2_H_4_ and ClO^−^ to produce two different products, which may lead to a fluorescence “on-off” strategy with different fluorescence signals. In Scheme 1, the synthesis process of probe **XBT-CN** is given. Firstly, at room temperature, cyclohexanone reacts with PBr_3_ in CH_2_Cl_2_ to give compound **1**. Compound **2** was obtained by refluxing 2-hydroxy-4-methoxybenzaldehyde with compound **1** in DMF. Then, there was a condensation reaction occurred between compound **2** and 2-benzothiazole acetonitrile (**3**) in the presence of the catalytic amount of piperidine, and the probe **XBT-CN** was obtained as a dark blue solid. The probe **XBT-CN** and related intermediates were confirmed by ^1^H NMR, ^13^C NMR, and HRMS (Figures S1–S12).

### 2.2. UV Response of **XBT-CN** to N_2_H_4_ and ClO^−^

**Figure 2:**
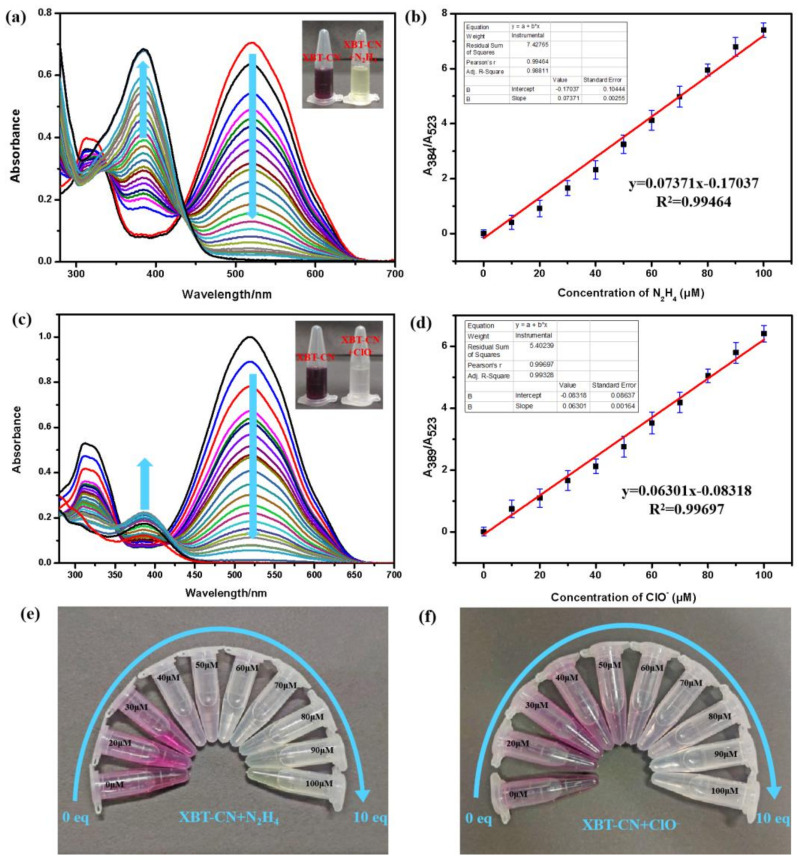
UV−vis absorption spectra of **XBT-CN** (10 μM) with the addition of various concentrations of N_2_H_4_ (0−100 μM) (**a**) and ClO^−^ (0−100 μM) (**c**) in CH_3_OH/PBS solution (8/2, *v*/*v*, pH 7.4). The linear relationship between the absorption intensity ratio of **XBT-CN** (10 μM) and the concentrations of N_2_H_4_ (0−100 μM) (**b**) and ClO^−^ (0−100 μM) (**d**). Photographs of probe **XBT-CN** in the presence of increasing concentrations of N_2_H_4_ (0−100 μM) (**e**) and ClO^−^ (0−100 μM) (**f**) under a sunlight lamp.

The sensing properties of probe **XBT-CN** for N_2_H_4_ and ClO^−^ in CH_3_OH/PBS solution (*v*/*v* = 8:2, pH = 7.4, 10 mM) were determined for the first time by UV-Vis and fluorescence spectra. As can be seen from Figure 2a, the UV-Vis spectra of **XBT-CN** (10 μM) have two absorption peaks at 312 nm and 523 nm, respectively. With the addition of N_2_H_4_ (0–100 μM), the maximum absorption peaks at 312 nm and 523 nm decreased, and the peak at 384 nm increased gradually. Simultaneously, the modena color of the **XBT-CN** solution gradually changes to yellow under sunlight, which can be observed by the naked eye (Figure 2a, inset). As shown in Figure 2c, we also investigated the UV-Vis absorption of ClO^−^ by **XBT-CN**. After ClO^−^ (0–100 μM) was added, the absorption peaks of **XBT-CN** at 312 nm and 523 nm gradually decreased with the peak at 389 nm increased, and the color of the **XBT-CN** solution changed from a modena color to colorless under sunlight, which was beneficial to naked-eye monitoring of ClO^−^ (Figure 2c, inset). Furthermore, we also studied the color changes of **XBT-CN** solution with the addition of different concentrations of N_2_H_4_ and ClO^−^ under sunlight (Figure 2e,f), and these results significantly show that the probe **XBT-CN** can provide visual detection of N_2_H_4_ and ClO^−^ by naked eyes.

### 2.3. Fluorescence Response of **XBT-CN** to N_2_H_4_ and ClO^−^

**Figure 3:**
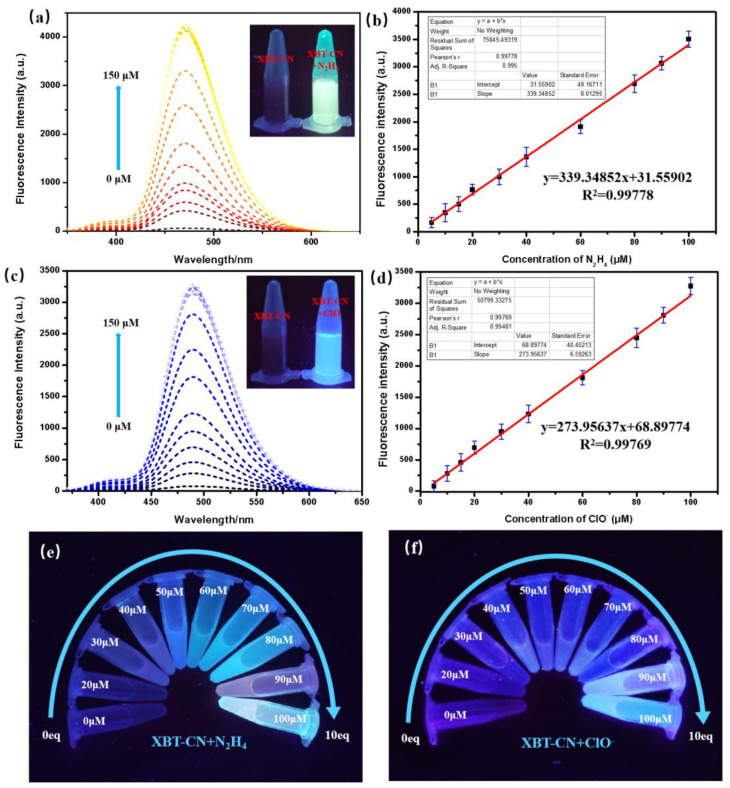
Fluorescence emission spectra of **XBT-CN** (10 μM) with addition of various concentrations of N_2_H_4_ (0−100 μM) (**a**) and ClO^−^ (0–100 μM) (**c**) in CH_3_OH/PBS solution (8/2, *v*/*v*, pH 7.4), λ_ex_ = 440 nm, λ_em_(N_2_H_4_) = 470 nm, λ_em_(ClO^−^) = 490 nm (Inset: the color fluorescence images of 10 μM **XBT-CN** in the absence and presence of 100 μM N_2_H_4_/ClO^−^ under illumination with a 365 nm UV lamp); The linear relationship between fluorescent intensity of **XBT-CN** (10 μM) and the concentrations of N_2_H_4_ (0−100 μM) (**b**) and ClO^−^ (0−100 μM) (**d**). Fluorescent photographs of probe **XBT-CN** in the presence of increasing concentrations of N_2_H_4_ (**e**) and ClO^−^ (**f**) under a 365 nm UV lamp.

The fluorescence response of **XBT-CN** (10 μM) in mixed solution (CH_3_OH/PBS, *v*/*v* = 8/2, pH = 7.4) with or without N_2_H_4_/ClO^−^ was also investigated. As shown in Figure 3a, **XBT-CN** had a weak fluorescence intensity at 470 nm. After adding different concentrations of N_2_H_4_ (0–100 μM), the fluorescence intensity at 470 nm increased gradually until it reached the maximum at 100 μM. This change made the fluorescence of **XBT-CN** change from quenched to luminous yellow, which was witnessed by the naked eye under a 365 nm lamp (Figure 3e). In addition, in the range of N_2_H_4_ concentration from 0 to 100 μM, the emission intensity has a good linear relationship with N_2_H_4_ concentration (R^2^ = 0.9978), and the LOD is 27 nM (Figure 3b). In addition, the responses of **XBT-CN** and ClO^−^ were also studied. As exhibited in Figure 3c, when different concentrations of ClO^−^ (0–100 μM) were added, the fluorescence intensity of **XBT-CN** increased at 490 nm, which inducing the fluorescence of **XBT-CN** changed from quenched to brilliant blue. This change was also observed under a 365 nm lamp (Figure 3f). In addition, in the range of 0–100 μM, the relationship curve between fluorescence intensity and ClO^−^ concentration (R^2^ = 0.9977) shows a good linear relationship, and the LOD is as low as 34 nM (Figure 3d). These results indicate that **XBT-CN** has high sensitivity in fluorescent detection of N_2_H_4_ and ClO^−^, providing a practical strategy for environmental safety assessment and biological sample detection.

### 2.4. Selectivity and Anti-Interference Performance of **XBT-CN**

**Figure 4:**
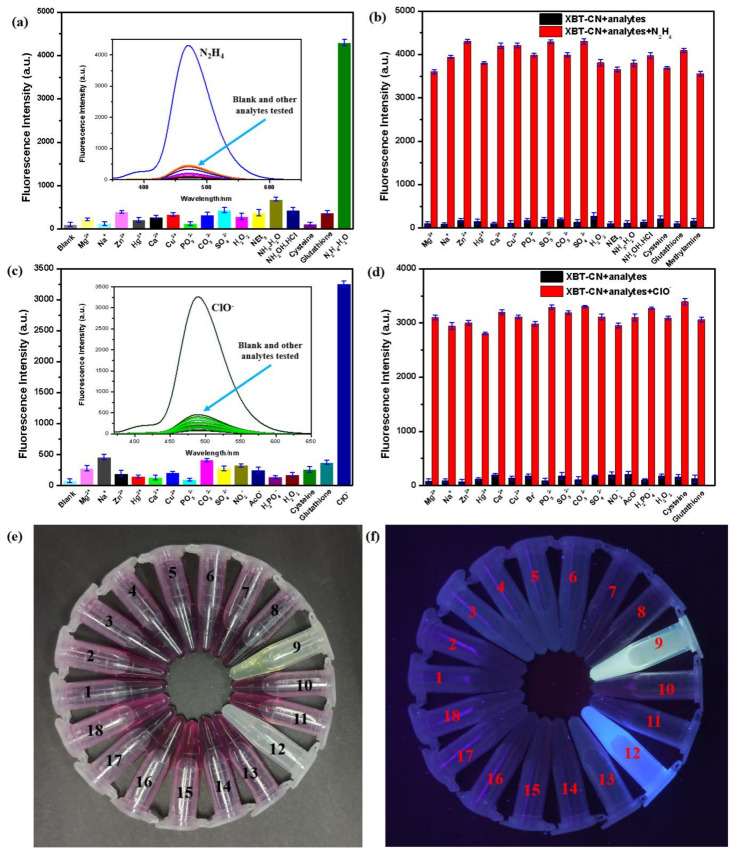
(**a**,**c**) Fluorescence performance of **XBT-CN** (10 μM) with the addition of various analytes (100 μM) in CH_3_OH/PBS solution (8/2, *v*/*v*, pH 7.4). (**b**,**d**) Competitive selectivity of **XBT-CN** (10 μM) to different analytes (100 μM) in CH_3_OH/PBS solution (8/2, *v*/*v*, pH 7.4). (**e**,**f**) The photographs of **XBT-CN** with the addition of various analytes in visible light and UV light. (1. **XBT-CN**, 2. Mg^2+^, 3. Na^+^, 4. Zn^2+^, 5. Hg^2+^, 6. Ca^2+^, 7 Cu^2+^, 8. PO_3_^2−^, 9. N_2_H_4_·H_2_O, 10. CO_3_^2−^, 11. SO_4_^2−^, 12. ClO^−^, 13. H_2_O_2_, 14. Cysteine, 15. Glutathione, 16. NEt_3_, 17. NH_3_·H_2_O, 18.NH_2_OH·HCl).

In order to evaluate the selectivity of **XBT-CN** to N_2_H_4_ and ClO^−^, the fluorescence performance of the probe in the presence of all kinds of interference was recorded. As shown in Figure 4a, the selectivity of probe **XBT-CN** to N_2_H_4_ was judged by using various analytes, such as anions, metal ions and amine. Except for N_2_H_4_, other analytes cannot cause obvious enhancement of fluorescence intensity. In addition, in the system where N_2_H_4_ coexists with other analytes (Figure 4b), the fluorescence response of **XBT-CN** to N_2_H_4_ was almost undisturbed. On the contrary, N_2_H_4_ significantly increased the fluorescence intensity of **XBT-CN** in CH_3_OH/PBS solution (70 times). We also obtained similar results, that is, **XBT-CN** showed high selectivity to ClO^−^. As described in Figure 4c,d, various analytes, such as anions, metal ions and other oxidants, did not lead to apparent enhancement of fluorescence intensity except for ClO^−^. These results demonstrate that **XBT-CN** has good specificity for the detection of N_2_H_4_ and ClO^−^ in actual environment and complex physical environment.

### 2.5. Effect of pH and Response Time

In the practical application of probes, pH value is an important influencing factor. The effect of pH on the fluorescence response of **XBT-CN** in the presence and absence of N_2_H_4_ and ClO^−^ was studied. As shown in Figure S13, in the range of pH 1.0–14.0, the fluorescence intensity of the probe itself has little change. This result suggests that the properties of **XBT-CN** are stable in a wide pH range and can be applied to detect the complex samples. Afterward, we recorded the fluorescence changes of **XBT-CN** treated with N_2_H_4_ or ClO^−^. When the probe was incubated with N_2_H_4_ or ClO^−^, the fluorescence intensity changed obviously in the range of pH 1.0–7.0, and reached the maximum in the range of pH 7.0–8.0. The fluorescence intensity under strong acidity in the pH range of 1 to 5 is weak, which may be the reason for the protonation of the analyte. Another reason may due to hydrazine’s strong alkalinity mainly existing in the form of [N_2_H_5_]^+^ under acidic conditions and loss of nucleophilicity. However, with the increase in pH value in the range of 9 to 14, the fluorescence intensity decreased obviously. This phenomenon may due to the nucleophilic attack of OH^−^ on **XBT-CN** under strong alkaline conditions, which disturb the nucleophilic attack on **XBT-CN** by N_2_H_4_ or ClO^−^. The results show that the probe **XBT-CN** has a good response to N_2_H_4_ and ClO^−^ in the range of pH 7.0–8.0, and can be hopeful for the detection of N_2_H_4_ and ClO^−^ in biological system.

With the above promising results in hand, the time-dependent fluorescence responses of **XBT-CN** toward N_2_H_4_ and ClO^−^ were further studied in CH_3_OH/PBS (*v*/*v* = 8:2, pH = 7.4) at room temperature. As revealed in Figure S14, there were almost no fluorescence intensity at both 470 nm and 490 nm in the solution when without N_2_H_4_ or ClO^−^. However, with the addition of N_2_H_4_, the fluorescence intensity of **XBT-CN** at 470 nm promptly enhanced and reached a plateau at 20 s. Meanwhile, the fluorescence intensity of **XBT-CN** at 490 nm increased gradually in the presence of ClO^−^ and became saturated at 15 s. These results strongly suggest that the rapid response probe **XBT-CN** can realized the real-time detection of N_2_H_4_ and ClO^−^. Further kinetic studies also showed that the calculated *pseudo*-first-order rate constant (*k’*) for detecting of N_2_H_4_ and ClO^−^ was 0.3656 min^−1^ and 0.3374 min^−1^, respectively (Figures S16 and S17). All the above results demonstrated that **XBT-CN** is a promising fluorescent probe, and could detect N_2_H_4_ and ClO^−^ in quantitative and an expressed speed by the fluorescence spectrometry method.

### 2.6. Sensing Mechanism Study of **XBT-CN** to N_2_H_4_ and ClO^−^

**Scheme 2:**
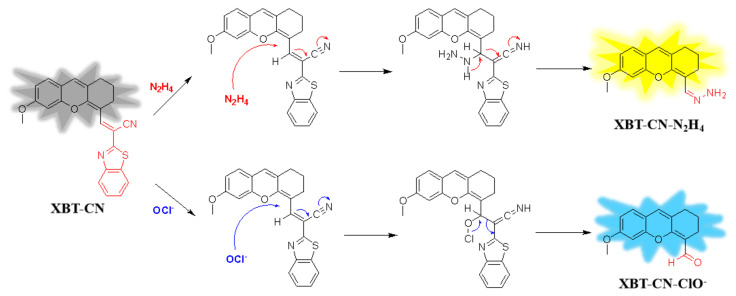
Proposed reaction scheme of sensor **XBT-CN** with N_2_H_4_ and ClO^−^.

For purpose of determine the reaction mechanism, we investigated the reaction of **XBT-CN** with N_2_H_4_ and ClO^−^ on the basis of HRMS analysis. HRMS spectra of **XBT-CN** in CH_3_OH, N_2_H_4_, and ClO^−^ for 30 min were recorded respectively. As shown in Figure S15A, **XBT-CN** (*ε* = 3.39 × 10^3^ M^−1^ cm^−1^, *Φ* = 0.002) itself showed a peak at *m*/*z* 398.1163. When **XBT-CN** (10 μM) is treated with N_2_H_4_ (100 μM), the original peak at *m*/*z* 398.1163 disappeared and a significant peak is observed at *m*/*z* 256.1286, which is consistent with the value estimated with response product **XBT-CN**-N_2_H_4_ (expected [M + H]^+^ at 256.1212, Figure S16B). This spectral change shows that a hydrazone derivative **XBT-CN**-N_2_H_4_ is formed through the nucleophilic addition reaction between the probe and N_2_H_4_, and the reaction mechanism is illustrated in Scheme 2. Furthermore, we also analyzed the reaction product **XBT-CN**-ClO^−^-adduct (Figure S16C). The product was separated and studied by HRMS. The peak at 242.1017 *m*/*z* is consistent with the expected formylation product (expected [M + H]^+^ 242.0943). The reasonable mechanism between **XBT-CN** and ClO^−^ is also described in Scheme 1. The C=C bond of **XBT-CN** was first attacked by ClO^−^, and then cleaved by ClO^−^ oxidation to form an aldehyde derivative (**XBT-CN**-ClO^−^). Thus it can be seen, these results prove that the products produced by the reaction of probe **XBT-CN** with N_2_H_4_ and ClO^−^ are expected **XBT-CN**-N_2_H_4_ (*ε* = 7.12 × 10^4^ M^−1^ cm^−1^, *Φ* = 0.46) and **XBT-CN**-ClO^−^ (*ε* = 9.74 × 10^4^ M^−1^ cm^−1^, *Φ* = 0.39), respectively.

**Figure 5:**
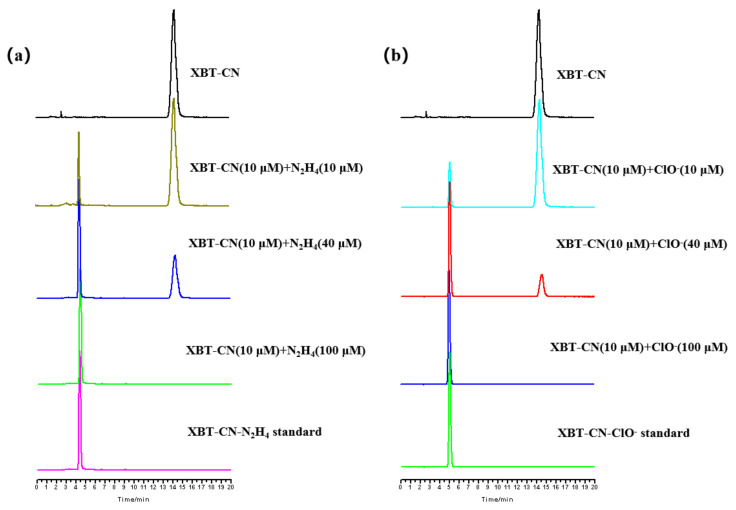
HPLC chromatograms of probe **XBT-CN** reacting with N_2_H_4_ (**a**) and ClO^−^ (**b**).

The proposed sensing process was further inspected via HPLC analysis. As is shown in Figure 5a, the probe **XBT-CN** displayed a main peak with retention times (t_R_) of 14.47 min. After the incubation of **XBT-CN** (10 μM) with N_2_H_4_ (10 μM), a new peak at 4.51 min appeared, which was corresponded to that of **XBT-CN**-N_2_H_4_. Incubating **XBT-CN** (10 μM) with high concentration of N_2_H_4_ (100 μM) leads to a chromatographic peak identical to that of synthetic **XBT-CN**-N_2_H_4_ standard, indicating the complete conversion of **XBT-CN** to **XBT-CN**-N_2_H_4_. In addition, when **XBT-CN** (10 μM) reacted with ClO^−^ (10 μM), a new peak at 4.51 min appeared, which was consistent with the peak of **XBT-CN**-ClO^−^ (Figure 5b). Furhtermore, **XBT-CN** (10 μM) reacting with high concentration of ClO^−^ (100 μM) leads to a chromatographic peak corresponding to that of synthetic **XBT-CN**-ClO^−^ standard, indicating the complete conversion of **XBT-CN** to **XBT-CN**-ClO^−^. These HPLC results evidently confirmed that the sensing mechanism which is consistent with the HRMS results.

**Figure 6:**
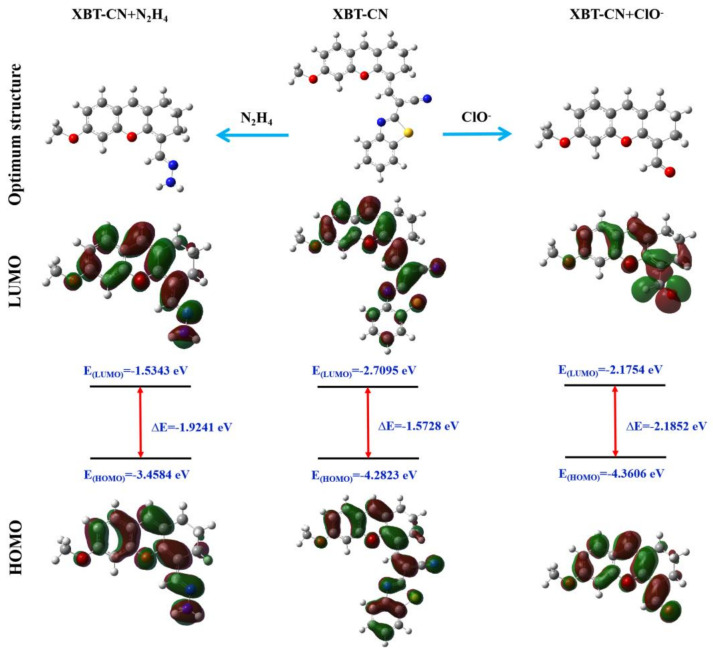
Optimum structure, HOMO−LUMO energy gaps of the orbitals for **XBT-CN** before and after reaction with N_2_H_4_ and ClO^−^ were calculated. Calculations were performed with the density functional theory method [B3LYP/6−31 G(d)] using Gaussian 09 program.

The interaction mechanism of **XBT-CN** with N_2_H_4_ and ClO^−^ was further confirmed by DFT calculations. The molecule structures of **XBT-CN** and the response products to N_2_H_4_ and ClO^−^ were calculated by density functional theory at B3LYP/6-31 G(d) level using Gaussian 09 program. As shown in Figure 6, when reacted with N_2_H_4_, the optimized configurations of **XBT-CN** and **XBT-CN**-N_2_H_4_ are acquired. Their energies of HOMO and LUMO orbits are also calculated. Furthermore, the energy gap of HOMO-LUMO orbit of **XBT-CN** was 1.5728 eV. After reacting with N_2_H_4_ and ClO^−^, the energy gaps of HOMO-LUMO orbit of **XBT-CN**-N_2_H_4_ and **XBT-CN**-ClO^−^ were 1.9241 eV and 2.1852 eV respectively, both higher than that of **XBT-CN**. This result may be because of the generation of C=N and C=O bond from the nucleophilic and oxidation reaction of C=C induced by N_2_H_4_ and ClO^−^, respectively. The above calculation results are completely in agreement with the spectral analysis, further demonstrating the proposed sensing mechanism of the probe **XBT-CN**.

### 2.7. Point-of-Care Detection of N_2_H_4/_ClO^−^ by Hydrogel Test Kit with Smartphone

**Figure 7:**
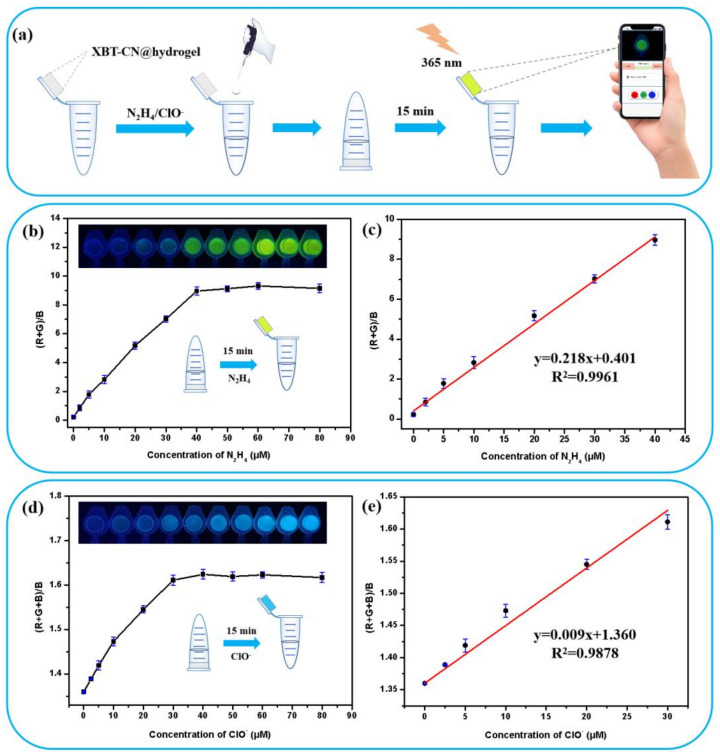
(**a**) Schematic diagram of **XBT-CN**@ agarose hydrogel sensing platform combined with a smartphone to detect N_2_H_4_ and ClO^−^. (**b**) The linear relationship between B/(R+G) and concentrations of N_2_H_4_ (0–80 μM). The illustration is photo of **XBT-CN**@ agarose hydrogel at 365 nm to identified different concentrations of N_2_H_4_. (**c**) The linear relationship between B/(R+G) and concentrations of N_2_H_4_ (0–30 μM). (**d**) The linear relationship between (R+G+B)/B and concentration of ClO^−^ (0–80 μM). The illustration is photo of **XBT-CN**@ agarose hydrogel at 365 nm to identified different concentrations of ClO^−^. (**e**) The linear relationship between (R+G+B)/B and concentration of ClO^−^ (0–30 μM).

To achieve the point-of-care detection of N_2_H_4_ and ClO^−^, a portable **XBT-CN**@hydrogel test kit was fabricated by placing agarose loaded with **XBT-CN** in a lid of centrifuge tube. The detection procedure for N_2_H_4_ and ClO^−^ by the portable test kit was displayed in Figure 7a. Moreover, the smartphone can photograph pictures of the fluorescence images and then exhibit values of R (red), G (green), and B (blue) to respond to different samples promptly. As could be seen from Figure 7b, with the addition of different concentrations of N_2_H_4_ or ClO^−^, the fluorescence colors of the hydrogel test kit displayed a range of changes under UV light. After that, the color-analysis smartphone app analyzed the colors and immediately output the corresponding values of RGB. Interestingly, the RGB values was interconnected with the concentration of N_2_H_4_ or ClO^−^. In the N_2_H_4_ titration experiment, there was a good linear relationship between (R+G)/B value and N_2_H_4_ concentration (R^2^ = 0.9961) in the range of 0–40 μM, and the LOD was calculated to be 19 nM (Figure 7c). Similarly, in the titration experiment of ClO^−^, there was also a good linear relationship existed between (R+G+B)/B value and ClO^−^ concentration (R^2^ = 0.9878) within the range of 0–30 μM, and the LOD was calculated to be 28 nM. The LODs are lower than previous fluorescence methods based on bulky instruments, revealing the excellent detection sensitivity of this portable test kit. Thus it can be seen, the **XBT-CN**@hydrogel test kit combined smartphone holds a great potential to be a portable tool for point-of-care detection of N_2_H_4_ and ClO^−^.

### 2.8. Application in Real Samples

**Figure 8:**
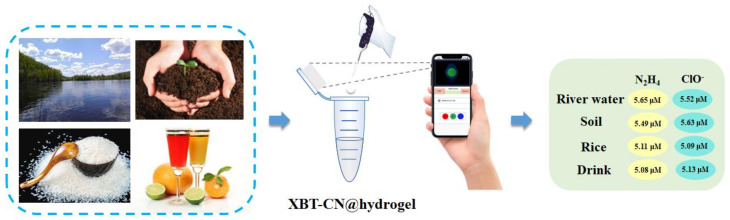
Schematic diagram of using the smartphone sensing platform to detect N_2_H_4_ and ClO^−^ in real samples.

Inspired by the above results, N_2_H_4_ and ClO^−^ in actual samples were detected by the **XBT-CN**@hydrogel test kit to demonstrate their practicability. We mainly focus on real samples in the environment (such as water and soil) and food samples. Environmental samples include soil (from cropland, wetlands, and sandland) and water (from tap water, lake water, and river water). Food samples include rice, flour, vegetables, and drinks. All the above species are might under the pressure of N_2_H_4_ or ClO^−^ risks. Firstly, the test solution of the actual sample was prepared, and then exogenous N_2_H_4_ and ClO^−^ with different concentrations (5.0 μM and 10.0 μM) were added. Then the sample solution was dropped into the hydrogel that was placed on the lid of the centrifuge tube and placed upside down for 15 min. Finally, the fluorescent color photos of the lids were photographed and input into the smartphone. According to the above linear regression equation, the levels of N_2_H_4_ and ClO^−^ in different samples were obtained (Figure 8). Obviously, the **XBT-CN**@hydrogel test kit could quantitatively detect N_2_H_4_ and ClO^−^ in real samples.

In order to further evaluate the practical application ability of the **XBT-CN**@hydrogel test kit in various practical samples, different concentrations of N_2_H_4_ and ClO^−^ in water, soil, and food were further determined by the probe **XBT-CN** in two methods. As shown in Tables S2–S7, the detection results are consistent with the actual addition of N_2_H_4_ or ClO^−^ in each sample by either the spectroscopic method or hydrogel test kit. Furthermore, the results showed that the recoveries ranged from 97% to 106%. The experimental results prove that **XBT-CN** can be used for the determination of N_2_H_4_ and ClO^−^ in various real samples and that the spectroscopic method and the hydrogel test kit have high accuracy and reliability for the quantitative determination of N_2_H_4_ and ClO^−^ in the environment and food.

### 2.9. Analysis of ClO^−^ in MPO/H_2_O_2_/Cl^−^ System

In order to evaluate the ability to detect ClO^−^ generated enzymatically employing MPO, the control experiment was conducted, as shown in Figure S18. After the addition of **XBT-CN** to the MPO/H_2_O_2_/Cl^−^ system, a rapid fluorescence intensity response was observed. The fluorescence growth for **XBT-CN** lasted for 5 min and then became steady. As depicted in Figure S18, the formation of product **XBT-CN-**ClO^−^ occurred only when MPO, NaCl, and H_2_O_2_ were present in the reaction mixture. The addition of catalase, which is responsible for the decomposition of hydrogen peroxide to water and oxygen, prevented the formation of ClO^−^, and thus oxidation of **XBT-CN** to **XBT-CN-**ClO^−^. The absence of any component necessary for the enzymatic production of HOCl resulted in the lack of formation of **XBT-CN-**ClO^−^. It was also proved that the **XBT-CN** probe is able to detect MPO activity in real time.

### 2.10. Role as Encryption Ink in Data Security

**Figure 9:**
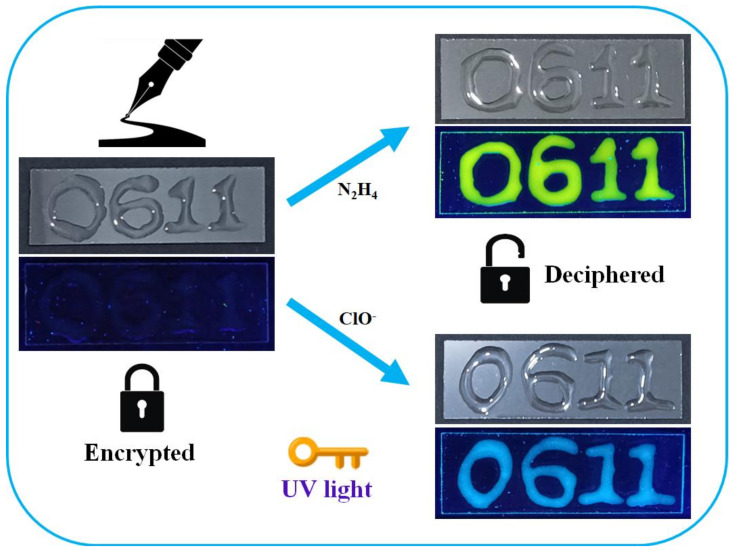
Naked-eye encryption and decryption of **XBT-CN@**hydrogel used as encryption ink on the glass plate observed in normal light and UV light, respectively, after contact with N_2_H_4_ and ClO^−^.

Encouraged by the strong fluorescence visual response of **XBT-CN**@hydrogel to N_2_H_4_/ClO^−^ accompanied by its excellent stability, liquidity, and injectability, we found that **XBT-CN**@hydrogel can be used as encryption ink in the field of information security. As shown in Figure 9, the number “0611” was written on a thin glass slide with **XBT-CN**@hydrogel. In the beginning, the numbers were invisible with weak fluorescence under UV light. However, the numbers can be illuminated immediately and send out luminous yellow fluorescence visualization in 3 s by spraying a N_2_H_4_ solution. Similarly, once the NaClO solution was applied, the number “0611” displayed a strong blue fluorescence. In the absence and presence of N_2_H_4_/ClO^−^, the **XBT-CN**@hydrogel always presented achromatous under sunlight. Therefore, **XBT-CN**@hydrogel has a broad prospect to be used as an encryption ink in the field of data security.

### 2.11. Fluorescence Imaging in Living Cells

**Figure 10:**
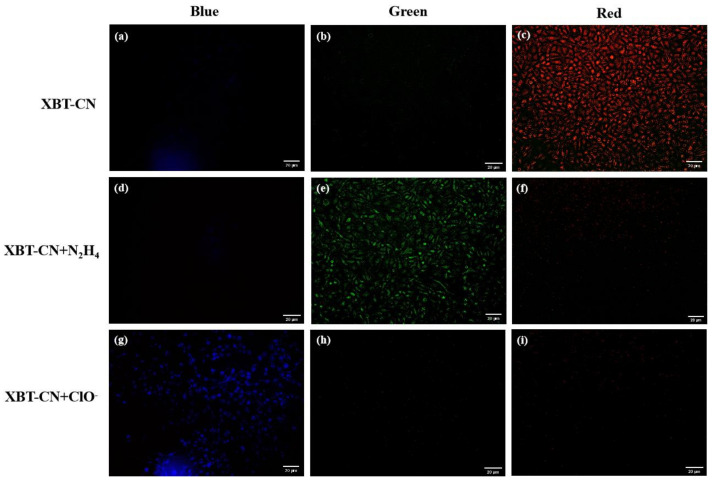
Fluorescence microscopy images of N_2_H_4_/ClO^−^ detection in GL261 cells using **XBT-CN** (20 μM). GL261 cells were incubated with **XBT-CN** for 2 h (**a**–**c**), and then further cultured for 2 h in the presence of N_2_H_4_ (80 μM) (**d**–**f**) or ClO^−^ (100 μM) (**g**–**i**) at blue channel (**a**,**d**,**g**); green channel (**b**,**e**,**h**); and red channel (**c**,**f**,**i**). The incubation temperature was 37 °C. Scale bar: 20 μm.

The above experiments initially proved that **XBT-CN** could be used to detect N_2_H_4_ and ClO^−^ in a complex system, and the detection ability of the **XBT-CN** probe in living cells was further investigated. Firstly, the toxicity of **XBT-CN** to GL261 cells (mouse glioma cells) was evaluated by standard MTT assay. After being treated with different concentrations of **XBT-CN** for 24 h, the cell survival rate was over 94%, as shown in Figure S19, which indicated that **XBT-CN** had low cytotoxicity and excellent biocompatibility in living cells. Afterward, the imaging ability of the probe to N_2_H_4_ and ClO^−^ in living cells was evaluated by fluorescence microscope. GL261 cells were incubated with **XBT-CN** (20 μM) for 2 h, and intracellular red fluorescence was observed (Figure 10c). **XBT-CN** treated cells were then incubated with N_2_H_4_ (80 μM) or ClO^−^ (100 μM) for 2 h. As exhibited in Figure 10e, the red fluorescence at the red channel was weakened, and the green fluorescence at the green channel was obvious, which indicated that the C=C double bond of **XBT-CN** was transformed into hydrazone by N_2_H_4_, which caused the green fluorescence to increase. On the other hand, we also obtained a clear blue fluorescence signal in cells under a fluorescence microscope, indicating that ClO^−^ can completely oxidize the C=C double bond of **XBT-CN** into aldehyde and induce the fluorescence of living cells to change from red to blue (Figure 10g). These results indicate that **XBT-CN** possesses low cytotoxicity and excellent biocompatibility and further can be used as a reliable and powerful tool for the detection and visualization of N_2_H_4_ and ClO^−^ in living cells.

## 3. Materials and Methods

### 3.1. Materials and Instruments

All chemicals and solvents were purchased from commercial sources and used without further purification. Solvents were purified by standard procedures. The water was purified by a Millipore filtration system. All chemical reactions were detected by thin-layer chromatography under 254 nm (or 365 nm) UV lamp. ^1^H and ^13^C NMR spectra were recorded on a WIPM 400 spectrometer using tetramethylsilane (TMS) as the internal standard. High-resolution mass (HRMS) spectra were recorded on a Waters LCT Premier XE spectrometer using standard conditions (ESI, 70 eV). All absorption spectrums were measured on a UV-1800 UV visible spectrophotometer. All fluorescence spectra were measured on an FL-4500 fluorescence spectrometer. The pH measurements were taken on a METTLER TOLEDO FiveEasy Plus pH meter. The cell imaging experiments were taken under an Olympus IX71 inverted fluorescence microscope.

### 3.2. Synthesis

As illustrated in Scheme 1, the probe **XBT-CN** was synthesized through a process including four steps. The structures of all the related intermediates and probe were confirmed by ^1^H NMR, ^13^C NMR, and HRMS spectra (Figure S1–S12).

#### 3.2.1. Synthesis of Compound **1**

The mixture of DMF (4.5 mL, 60.0 mmol) and CH_2_Cl_2_ (30 mL) was kept at 0 °C. Then PBr_3_ (3.8 mL, 60.0 mmol) was slowly added into the mixture and stirred for 1.0 h at 0 °C. After that, cyclohexanone (1.7 mL, 20.0 mmol) was dropwise added into the mixture and stirred for 16 h at room temperature. After the reaction was completed, the mixture was slowly poured into ice water (100 mL), and NaHCO_3_ powder was slowly added to the mixture until the pH = 7. The solution was extracted with CH_2_Cl_2_, and the organic layer was dried with anhydrous Na_2_SO_4_ for 8 h. Finally, the solvent was evaporated to give **1** as a yellow oil (3.20 g, 85 % yield). ^1^H NMR(400 MHz, DMSO-d_6_) δ (TMS, ppm): 1.72–1.69 (m, 2H), 1.77–1.79(t, *J* = 4.0 Hz, 2H), 2.29–2.31(t, *J* = 4.0 Hz, 2H), 2.77 (m, 2H), and 10.04(s, 1H). ^13^C NMR (100 MHz, DMSO-d_6_) δ (TMS, ppm): 21.09, 24.26, 25.00, 38.83, 135.29, 143.59, 193.74. EI(+)-HRMS(m/z): [M]^+^ calcd. for C_7_H_9_BrO: 187.9837, found: 187.9831.

#### 3.2.2. Synthesis of Compound **2**

Compound **1** (0.47 g, 2.5 mmol) and 2-hydroxy-4-methoxybenzaldehyde (0.32 g, 2.0 mmol) were dissolved in DMF (10 mL), and Cs_2_CO_3_ (1.89 g, 6.0 mmol) were added to the mixture subsequently. The reaction mixture was stirred for 12 h at room temperature and then filtered and concentrated. The concentration was dissolved in CH_2_Cl_2_ (50 mL) and was washed with pure water (30 mL × 3). The organic layer was dried with anhydrous Na_2_SO_4_ for 8 h and then filtered. Afterward, the solvent was concentrated, and the obtained crude product was further purified by silica gel column chromatography (PE/EA, *v*/*v* = 15:1) to afford **2** as a yellow solid with a yield of 77% (0.37 g). ^1^H NMR (400 MHz, DMSO-d_6_) δ (TMS, ppm): 1.71–1.74 (t, *J* = 8.0 Hz, 2H), 2.44–2.47 (t, *J* = 8.0 Hz, 2H), 2.57 (m, 2H), 3.85 (s, 3H), 6.65–6.68 (q, 3H), 7.09–7.10 (d, *J* = 8.0 Hz, 1H), 10.32 (s, 1H). ^13^C NMR (100 MHz, DMSO-d_6_) δ (TMS, ppm): 20.42, 21.53, 29.91, 55.66, 100.50, 110.85, 112.59, 114.69, 126.59, 126.84, 127.46, 153.38, 160.77, 161.39, 187.60. ESI (+)-HRMS (m/z): [M+H]^+^ calcd. for C_15_H_14_O_3_: 242.0943, found: 242.1017.

#### 3.2.3. Synthesis of Compound **3**

The mixture of *o*-aminophenol (0.18 g, 1.0 mmol) and malononitrile (0.26 g, 1.2 mmol) in EtOH (10 mL) was refluxed for 9 h. Furthermore, the reaction solution was concentrated after the reaction was completed. Then the concentration was further purified by column chromatography (PE/EA, *v*/*v* = 20:1) to afford **3** as a white solid (0.15 g, 88 % yield). ^1^H NMR (400 MHz, CDCl_3_-d_1_) δ (TMS, ppm): 4.26 (s, 2H), 7.45–7.53 (m, 1H), 7.55–7.57 (t, *J* = 4.0 Hz, 1H), 7.92–7.93 (d, *J* = 4.0 Hz, 1H), 8.07–8.08 (d, *J* = 8.0 Hz, 1H). ^13^C NMR (100 MHz, CDCl_3_-d_1_) δ (TMS, ppm): 23.23, 114.89, 121.76, 123.44, 126.01, 126.75, 135.49, 152.88, 158.25. ESI (+)-HRMS (m/z): [M+H]^+^ calcd. for C_9_H_6_N_2_S: 174.0252, found: 174.0320.

#### 3.2.4. Synthesis of Compound **XBT-CN**

Firstly, compound **2** (0.18 g, 1.0 mmol) and compound **3** (0.24 g, 1.2 mmol) were dissolved in 10 mL EtOH. Later, piperidine (0.1 mL) was dropped into the mixture, and the solution was refluxed for 12 h. Furthermore, the reaction solution was concentrated after the reaction was completed. Then the concentration was further purified by column chromatography (PE/EA, *v*/*v* = 30:1) to afford **XBT-CN** as a blue solid (0.21 g, 53 % yield). ^1^H NMR (400 MHz, DMSO-d_6_) δ (TMS, ppm): 1.28 (s, 1H), 1.86 (s, 2H), 2.58 (s, 2H), 3.03 (s, 2H), 3.88 (s, 3H), 6.66–6.78 (t, *J* = 4.0 Hz, 3H), 7.08–7.09 (d, *J* = 4.0 Hz, 1H), 7.35 (s, 1H), 7.48 (s, 1H), 7.85–7.86 (d, *J* = 4.0 Hz, 1H), 8.03–8.04 (d, *J* = 4.0 Hz, 1H), 8.58 (s, 1H). ^13^C NMR (100 MHz, DMSO-d_6_) δ (TMS, ppm): 20.89, 25.72, 29.47, 55.47, 55.77, 97.37, 100.71, 109.91, 111.28, 115.16, 118.83, 121.32, 122.80, 124.88, 126.42, 127.23, 13471, 140.46, 158.12, 156.42, 161.58, 166.00. ESI (+)-HRMS (m/z): [M+H]^+^ calcd. for C_24_H_18_N_2_O_2_S: 398.1089, found: 398.1163.

### 3.3. Spectroscopic Measurements

Stock solution of the probe **XBT-CN** (1 mM) were prepared in CH_3_OH. Stock solutions of N_2_H_4_, ClO^−^, and other interfering analytes (10 mM) were all prepared in ultrapure water. All the fluorescence measurements were performed by scanning the spectra in the range of 200 nm and 700 nm and excited at the wavelength of 440 nm. The slit widths for both excitation and emission spectra were 5 nm. All fluorescence spectra were excited at 440 nm and acquired in CH_3_OH/PBS solution (*v*/*v* = 8:2, pH = 7.4, 10 mM) at room temperature.

### 3.4. Determination of the Fluorescence Quantum Yield

The fluorescence quantum yields of **XBT-CN**, **XBT-CN**-N_2_H_4_, and **XBT-CN**-ClO^−^ were determined with quinine sulfate (*Φ* = 0.54 in 0.1 M H_2_SO_4_) as a fluorescence standard. The quantum yields were calculated using the following equation:

*Φ*_r_ = *Φ*_s_(F_r_A_s_/F_s_A_r_)(n_r_/n_s_)^2^

*Φ*_r_ and *Φ*_s_ denote the quantum yield of the samples and standard sample, respectively. F, A, and n denote the region of the emission band, absorbance, and refractive index of the solvent, respectively. Subscripts s and r refer to the standard and the unknown, respectively.

### 3.5. Selectivity Study

Selectivity experiments were conducted to investigate the specificity of the probe **XBT-CN** toward N_2_H_4_ and ClO^−^ over other interfering analytes such as cations (Mg^2+^, Na^+^, Zn^2+^, Hg^2+^, Ca^2+^, Cu^2+^), anions (Br^−^, SO_4_^2−^, NO_2_^−^, CO_3_^2−^, PO_3_^2−^, SO_4_^2−^, AcO^−^, H_2_PO_4_^−^), amino acids (cysteine, glutathione), amines (NEt_3_, NH_3_·H_2_O, NH_2_OH·HCl) and oxidizing substance (H_2_O_2_). Stock solutions of these interfering analytes (10 mM) were prepared in CH_3_OH or ultrapure water and then diluted with CH_3_OH/PBS solution (*v*/*v* = 8:2, pH = 7.4, 10 mM). The final concentration of interfering analytes was 100 μM while the concentration of N_2_H_4_ and ClO^−^ was 10 μM, respectively.

### 3.6. HPLC Analysis

HPLC analysis was carried out on a Waters 2695 Alliance system (Milford, MA, USA) equipped with a quaternary solvent delivery system, a column oven, an auto-sampler, and a photodiode array detector. The analytes were separated with a C18 column (Welchrom, 150 mm × 4.6 mm, 5.0 μm). The flow rate was 1.0 mL/min. The eluent components were water (A) and acetonitrile (B). The mobile phase gradient was as follows: the proportion of Phase B increased from 10 to 80% over 10 min at the flow rate of 1.0 mL/min. The detection wavelength was set at 365 nm.

### 3.7. Density Functional Theory

According to the basis set of B3LYP/6-31 G(d) [46,47,48,49], after responding to N_2_H_4_ and ClO^−^, the geometrical configuration of the probe **XBT-CN** in the ground state and excited state were respectively calculated and optimized by density functional theory (DFT). Moreover, DFT can be applied to calculate the electron cloud distribution and energy gaps of the highest occupied molecular orbital (HOMO) and the lowest unoccupied molecular orbital (LUMO) state.

### 3.8. Preparation of the **XBT-CN**@Hydrogel Portable Test Kit

Firstly, agarose (0.25 g) was dissolved in 10 mL of ultrapure water and heated until it was completely dissolved, obtaining a hydrogel with an agarose content 2.5% (*w*/*v*). Secondly, 20 μL of **XBT-CN** (10 μM) was added to the hydrogel and stirred vigorously for 1 h. Then 200 μL above mixture was poured into the centrifuge tube lid and cooled at room temperature for the sake of using the tube as a portable **XBT-CN**@hydrogel test kit. Subsequently, N_2_H_4_ or ClO^−^ with different concentrations was added to the centrifuge tube. After that, the centrifuge tube was turned upside down for 15 min at room temperature to allow N_2_H_4_ or ClO^−^ diffuse into the hydrogel to react with **XBT-CN** completely. To observe the color change of the hydrogel, the centrifuge tube was turned upside down again, and the snap cap was opened. Fluorescence images of the hydrogel in the lid under a 365 UV light were recorded by smartphone and analyzed by a color-analysis app. Finally, the N_2_H_4_ or ClO^−^ concentration can be calculated according to the proportional relationship between them and RGB values.

### 3.9. Detection of N_2_H_4_ and ClO^−^ in Real Samples by Hydrogel Test Kit

For the purpose of verifying the practicability of the **XBT-CN**@hydrogel sensor combined with a smartphone, we selected several practical samples, such as water, soil, and food. Water samples were collected from the Hebei Technology University Campus (tap water, lake water), river water was collected from the Minxin River in Shijiazhuang, and industrial wastewater was collected from a pharmaceutical enterprise in Shijiazhuang. Both soil samples from cropland, wetland, and sandland were collected in Shijiazhuang. Food samples (rice, flour, beer, and cabbage) were purchased from the Beiguo supermarket in Shijiazhuang. All the real samples were pre-treated before the measurements. Water samples were standing placed for 12 h and then filtered. Each kind of soil sample (1.0 g) was added into pure water (5 mL), stirred for 12 h, and then filtered. Food samples like solid food other than drinks were firstly digested (refer to the reported pretreatment procedures) [50,51,52]. The solid food samples (1.0 g each) were added into 0.5% NaOH solution (*m*/*m*, 10 mL), stirred overnight at room temperature, and kept refluxed for 5 h. Then the mixture was cooled to room temperature and filtered. Afterward, the HCl solution (1.0 mol/L) was dropped into the filtrate to adjust the pH to 7.0–8.0. In addition, the samples of drinks were directly adjusted to pH 7.0–8.0 by HCl solution. The procedures and conditions used for the determination of N_2_H_4_ and ClO^−^ in real samples were the same as the above Section 3.6.

## 4. Conclusions

A novel dual-response fluorescence probe **XBT-CN** has been successfully developed for the detection of N_2_H_4_ and ClO^−^ simultaneously. In the nucleophilic reaction of C=C bond in **XBT-CN**, N_2_H_4_ induced the probe to produce a hydrazone structure with an obvious fluorescence change from quenched to luminous yellow. Meanwhile, It has been shown that ClO^−^ can oxidize C=C double bond to form an aldehyde derivative with obvious fluorescence change from quenched to brilliant blue. The probe **XBT-CN** can be used to quantitatively determine N_2_H_4_ and ClO^−^ with a rapid response time (within 20 s) and high sensitivity. The LOD of N_2_H_4_ and ClO^−^ is 27 nM and 34 nM, respectively. It has good selectivity in a complex physiological environment. It is worth noting that we have fabricated a portable hydrogel test kit integrated with dual-emission **XBT-CN** for point-of-care detection of N_2_H_4_ and ClO^−^ in environmental and food samples. Attractively, **XBT-CN**@hydrogel can also be employed as an encryption ink for information security applications. In addition, the results of living cell imaging showed that **XBT-CN** had low cytotoxicity and excellent biocompatibility and could recognize and visualize N_2_H_4_ and ClO^−^ in living cells. Overall, the neoteric dual-response fluorescence probe **XBT-CN** successfully realizes the simultaneous and point-of-care detection of N_2_H_4_ and ClO^−^ in various fields, such as environment, food, information security, and biological imaging.

## Data Availability

The data that support the findings of this study are available from the corresponding author upon reasonable request.

## Supplementary Materials

The following supporting information can be downloaded at: https://www.mdpi.com/article/10.3390/molecules28093896/s1. Table S1: Comparison of fluorescent probes for N_2_H_4_ and ClO^−^; Figure S1: ^1^H NMR spectrum of compound **1** in DMSO^−^d_6_; Figure S2: ^13^C NMR spectrum of probe 1 in DMSO-d6; Figure S3: HRMS spectrum of compound **1** in CH_3_OH; Figure S4: ^1^H NMR spectrum of compound **2** in DMSO-d6; Figure S5: ^13^C NMR spectrum of probe 2 in DMSO-d6; Figure S6: HRMS spectrum of compound **2** in CH_3_OH; Figure S7: ^1^H NMR spectrum of compound **3** in CDCl_3_-d1; Figure S8: ^13^C NMR spectrum of probe 3 in CDCl_3_-d1; Figure S9: HRMS spectrum of compound **3** in CH_3_OH; Figure S10: ^1^H NMR spectrum of probe XBT-CN in DMSO-d6; Figure S11: ^13^C NMR spectrum of probe **XBT-CN** in DMSO-d6; Figure S12: HRMS spectrum of probe **XBT-CN** in CH_3_OH; Figure S13: Effect of pH on the fluorescence intensity of **XBT-CN**; Figure S14: Time dependent fluorescence spectra of **XBT-CN** with added N_2_H_4_ and ClO^−^; Figure S15: HRMS spectrum of **XBT-CN** upon addition of N_2_H_4_ and ClO^−^; Figures S16 and S17: Pseudo-first-order kinetic plot; Tables S2–S7: Determination of N_2_H_4_ and ClO^−^ in real samples.; Figure S18: Time-dependent of fluorescence changes of **XBT-CN** in MPO/H_2_O_2_/Cl^−^; Figure S19: Viability of GL261 cells were treated with various concentrations of **XBT-CN** [31,34,36,37,39,42,43,44,53,54,55].

## References

[1] Serov A., Kwak C. (2010). Direct hydrazine fuel cells: A review. Appl. Catal. B Environ..

[2] Ramachandraih C.T., Subramanyam N., Bar K.J., Baker G., Yeragani V.K. (2011). Antidepressants: From MAOIs to SSRIs and more. Indian J. Psychiatr..

[3] Nguyen H.N., Chenoweth J.A., Bebarta V.S., Albertson T.E., Nowadly C.D. (2021). The toxicity, pathophysiology, and treatment of acute hydrazine propellant exposure: A systematic review. Mil. Med..

[4] Chen H.Y., Albertson T.E., Olson K.R. (2016). Treatment of drug-induced seizures. Br. J. Clin. Pharmacol..

[5] Sinha B.K., Mason R.P. (2014). Biotransformation of hydrazine dervatives in the mechanism of toxicity. J. Drug Metabol. Toxicol..

[6] Finkelstein D.A., Imbeault R., Garbarino S., Rou L., Guay D. (2016). Trends in catalysis and catalyst cost effectiveness for N_2_H_4_ fuel cells and sensors: A rotating disk electrode (RDE) study. J. Phys. Chem. C.

[7] Qin H.Y., Liu Z.X., Yin W.X., Zhu J.K., Li Z.P. (2008). Effects of hydrazine addition on gas evolution and performance of the direct borohydride fuel cell. J. Power Sources.

[8] Nobari N., Behboudnia M., Maleki R. (2016). Palladium-free electroless deposition of pure copper film on glass substrate using hydrazine as reducing agent. Appl. Surf. Sci..

[9] Niemeier J.K., Kjell D.P. (2013). Hydrazine and aqueous hydrazine solutions: Evaluating safety in chemical processes. Org. Process Res. Dev..

[10] Ioannidou H.A., Koutentis P.A. (2009). The conversion of Isothiazoles into pyrazoles using hydrazine. Tetrahedron.

[11] Aigner B.A., Darsow U., Grosber M., Ring J., Plőtz S.G. (2010). Multiple basal cell carcinomas after long-term exposure to hydrazine: Case report and review of the literature. Dermatology.

[12] Vivekanandan P., Gobianand K., Priya S., Vijayalakshmi P., Karthikeyan S. (2007). Protective effect of picroliv against hydrazine-induced hyperlipidemia and hepatic steatosis in rats. Drug Chem. Toxicol..

[13] Liu K., Huang S.J., Li T.R., Sun J., Fan L., Wang X.F., Li H.X., Li Y.J., Zhang W., Yang Z.Y. (2022). A benzocoumarin-based fluorescent probe for highly specific ultra-sensitive fast detecting endogenous/exogenous hypochlorous acid and its applications. J. Photoch. Photobiol. A Chem..

[14] Nguyen K.H., Hao Y.Q., Zeng K., Fan S.N., Li F., Yuan S.K., Ding X.J., Xu M.T., Liu Y.N. (2018). A benzothiazole-based fluorescent probe for hypochlorous acid detection and imaging in living cells. Spectrochim. Acta.

[15] Zhu B.C., Wu L., Zhang M., Wang Y.W., Liu C.Y., Wang Z.K., Duan Q.X., Jia P. (2018). A highly specific and ultrasensitive near-infrared fluorescent probe for imaging basal hypochlorite in the mitochondria of living cells. Biosens. Bioelectron..

[16] Swierczyńska M., Słowinski D., Grzelakowska A., Szala M., Romanski J., Pierzchała K., Siarkiewicz P., Michalski R., Podsiadły R. (2021). Selective, stoichiometric and fast-response fluorescent probe based on 7-nitrobenz-2-oxa-1,3-diazole fluorophore for hypochlorous acid detection. Dye. Pigment..

[17] Wu W.L., Zhao Z.M., Dai X., Su L., Zhao B.X. (2016). A fast-response colorimetric and fluorescent probe for hypochlorite and its real applications in biological imaging. Sens. Actuator B Chem..

[18] Zhao X.J., Jiang Y.R., Chen Y.X., Yang B.Q., Li Y.T., Liu Z.H., Liu C. (2019). A new “off-on” NIR fluorescence probe for determination and bio-imaging of mitochondrial hypochlorite in living cells and zebrafish. Spectrochim. Acta.

[19] Yan L., Hu C., Li J. (2018). A fluorescence turn-on probe for rapid monitoring of hypochlorite based on coumarin Schiff base. Anal. Bioanal. Chem..

[20] Huo F.J., Zhang J.J., Yang Y.T., Chao J.B., Yin C.X., Zhang Y.B., Chen T.G. (2012). A fluorescein based highly specific colorimetric and fluorescent probe for hypochlorites in aqueous solution and its application in tap water. Sens. Actuator B Chem..

[21] Chan C.M., Zhang W.N., Xue Z.L., Fang Y.Y., Qiu F.X., Pan J.M., Tian J.W. (2022). Near-infrared photoacoustic probe for reversible imaging of the ClO^−^/GSH redox cycle in vivo. Anal. Chem..

[22] Zhang Y.R., Liu Y., Feng X., Zhao B.X. (2017). Recent progress in the development of fluorescent probes for the detection of hypochlorous acid. Sens. Actuator B Chem..

[23] McAdam K., Kimpton H., Essen S., Davis P., Vas C., Wright C., Porter A., Rodu B. (2015). Analysis of hydrazine in smokeless tobacco products by gas chromatography-mass spectrometry. Chem. Cent. J..

[24] Karimi-Maleh H., Moazampour M., Ensafi A.A., Mallakpour S., Hatami M. (2014). An electrochemical nanocomposite modified carbon paste electrode as a sensor for simultaneous determination of hydrazine and phenol in water and wastewater samples. Environ. Sci. Pollut. Res. Int..

[25] Oh J.A., Shin H.S. (2017). Simple determination of hydrazine in waste water by headspace solid-phase micro extraction and gas chromatography-tandem mass spectrometry after derivatization with trifluoro pentanedione. Anal. Chim. Acta.

[26] Sharma S., Ganeshan S.K., Pattnaik P.K., Kanungo S., Chappanda K.N. (2020). Laser induced flexible graphene electrodes for electrochemical sensing of hydrazine. Mater. Lett..

[27] Tang X., Zhu Z., Wang Y., Han J., Ni L., Zhang H.Q., Li J., Mao Y.L. (2018). A cyanobiphenyl based fluorescent probe for rapid and specific detection of hypochlorite and its bio-imaging applications. Sens. Actuator B Chem..

[28] Jiang Q., Wang Z.L., Li M.X., Song J., Yang Y.Q., Xu X., Xu H.J., Wang S.F. (2020). A novel nopinone-based fluorescent probe for colorimetric and ratiometric detection of hypochlorite and its applications in water samples and living cells. Analyst.

[29] Zhang P.S., Wang H., Hong Y.X., Yu M.L., Zeng R.J., Long Y.F., Chen J. (2018). Selective visualization of endogenous hypochlorous acid in zebrafish during lipopolysaccharide-induced acute liver injury using a polymer micelles-based ratiometric fluorescent probe. Biosens. Bioelectron..

[30] Ren M.G., Li Z.H., Deng B.B., Wang L., Lin W.Y. (2019). Single fluorescent probe separately and continuously visualize H_2_S and HClO in lysosomes with different fluorescence signals. Anal. Chem..

[31] Zhu M.Q., Zhao Z.Y., Huang Y., Fan F.G., Wang F., Li W.L., Wu X.W., Hua R.M., Wang Y. (2021). Hydrazine exposure: A near-infrared ICT-based fluorescent probe and its application in bioimaging and sewage analysis. Sci. Total Environ..

[32] Tang C., Tong H.J., Liu B., Wang X.N., Jin Y.S., Tian E.L., Wang F. (2022). Robust ERα-targeted near-infrared fluorescence probe for selective hydrazine imaging in breast cancer. Anal. Chem..

[33] Wang L.Y., Xin S.Q., Xie F.R., Ran X.G., Tang H., Cao D.R. (2022). A novel windmill-shaped AIE-active pyrrolopyrrole cyanine: Design, synthesis and efficient hydrazine detection. J. Mater. Chem. C.

[34] Li D.H., Liu L., Yang H.G., Ma J., Wang H.L., Pan J.M. (2022). A novel dual-response triphenylamine-based fluorescence sensor for special detection of hydrazine in water. Mat. Sci. Eng. B.

[35] Zhang K., Huang Y., Shen Y.J., Ma S., Chen T.T. (2021). Combination of imine bond and samarium emitter enables turn-off fluorescence detection of hydrazine in vapor and water samples. Talanta.

[36] Huang X.H., Luo T.C., Zhang C., Li J.R., Jia Z.J., Chen X.L., Hu Y.J., Huang H. (2022). Dual-ratiometric fluorescence probe for viscosity and hypochlorite based on AIEgen with mitochondria-targeting ability. Talanta.

[37] Xu X.H., Ding H.C., Zhang Q., Liu G., Pu S.Z. (2022). A ratiometric fluorescent probe with an extremely large emission shift for detecting ClO^−^ and its application in test strips and cell imaging. Dye. Pigment..

[38] Zeng C.H., Xu Z.Y., Song C., Qin T.Y., Jia T.H., Zhao C., Wang L., Liu B., Peng X.J. (2023). Naphthalene-based fluorescent probe for on-site detection of hydrazine in the environment. J. Hazard. Mater..

[39] Li N.N., Gao Y.E., Xu X.Y., Qiu P., Gao Y., Yan M., Zhang Q., Lin W.Y., Xing Z.Y., Zong Z.A. (2023). Fluorescence probe with aggregation induced emission effect and application for colormetric detection of endogenous and exogenous hypochlorite. Dye. Pigment..

[40] Zhong X.L., Yang Q., Chen Y.S., Jiang Y.L., Dai Z.H. (2020). Aggregation-induced fluorescence probe for hypochlorite imaging in mitochondria of living cells and zebrafish. J. Mater. Chem. B.

[41] Zhu B.T., Wu X.L., Rodrigues J., Hu X.C., Sheng R.L., Bao G.M. (2021). A dual-analytes responsive fluorescent probe for discriminative detection of ClO^−^ and N_2_H_4_ in living cells. Spectrochim. Acta.

[42] Wang L., Pan Q., Chen Y., Ou Y.F., Li H.Y., Li B.W. (2020). A dual-response ratiometric fluorescent probe for hypochlorite and hydrazine detection and its imaging in living cells. Spectrochim. Acta.

[43] Das S., Patra L., Das P.P., Ghoshal K., Gharami S., Walton J.W., Bhattacharyyae M., Mondal T.K. (2022). A new ratiometric switch “two-way” detects hydrazine and hypochlorite via a “dye-release” mechanism with a PBMC bioimaging study. Phys. Chem. Chem. Phys..

[44] He X.J., Deng Z.A., Xu W., Li Y.H., Xu C.C., Chen H., Shen J.L. (2020). A novel dual-response chemosensor for bioimaging of exogenous/endogenous hypochlorite and hydrazine in living cells, pseudomonas aeruginosa and zebrafish. Sens. Actuator B Chem..

[45] Qiu L.M., Wan J.Y., Lu Y.H., Zhang P.T., Qin D.S., Yan J., Xiao H.B. (2022). A dual-site colorimetric fluorescent probe for rapid detection of hydrazine/hypochlorite and its application in two-photon fluorescent bioimaging. Results Chem..

[46] Stephens P.J., Devlin F.J., Chabalowski C.F., Frisch M.J. (1994). Ab initio calculation of vibrational absorption and circular dichroism spectra using density functional force fields. J. Phys. Chem..

[47] Rassolov V.A., Ratner M.A., Pople J.A., Redfern P.C., Curtiss L.A. (2001). 6-31G* basis set for third-row atoms. J. Comput. Chem..

[48] Frisch M.J., Trucks G.W., Schlegel H.B., Scuseria M.A., Robb J.R., Cheesman G., Scalmani V., Petersson G.A., Nakatsuji H., Li M. (2009). Gaussian 09, Revision C. 01.

[49] Zhao Y., Truhlar D.G. (2008). The M06 suite of density functionals for main group thermochemistry, thermochemical kinetics, noncovalent interactions, excited states, and transition elements: Two new functionals and systematic testing of four M06-class functionals and 12 other functionals. Theor. Chem. Acc..

[50] Qi Y.L., Chen J., Zhang B., Li H., Li D.D., Wang B.Z., Yang Y., Zhu H.L. (2020). A turn-on fluorescent sensor for selective detection of hydrazine and its application in *Arabidopsis thaliana*. Spectrochim. Acta.

[51] Guo Z.R., Niu Q.F., Yang Q.X., Li T.D., Wei T., Yang L., Chen J.B., Qin X.Y. (2020). New “naked-eye” colori/fluorimetric “turn-on” chemosensor: Ultrafast and ultrasensitive detection of hydrazine in similar to 100% aqueous solution and its bio-imaging in living cells. Anal. Chim. Acta.

[52] Wang J.L., Wang H., Yang S.X., Tian H.Y., Liu Y.G., Hao Y.F., Zhang J., Sun B.G. (2018). A fluorescent probe for sensitive detection of hydrazine and its application in red wine and water. Anal. Sci..

[53] Mu S., Gao H., Li C., Li S.S., Wang Y.Y., Zhang Y., Ma C.M., Zhang H.X., Liu X.Y. (2021). A dual-response fluorescent probe for detection and bioimaging of hydrazine and cyanide with different fluorescence signals. Talanta.

[54] Chen R., Shi G.J., Wang J.J., Qin H.F., Zhang Q., Chen S.J., Wen Y.H., Guo J.B., Wang K.P., Hu Z.Q. (2021). A highly-sensitive “turn on” probe based on coumarin β-diketone for hydrazine detection in PBS and living cells. Spectrochim. Acta.

[55] Zhang C.L., Li X.L., Jiang Y.H., Zhang Y.N., Xie Y.X., Sun Y.D., Liu C. (2022). A super large Stokes shift ratiometric fluorescent probe for highly selective sensing of ClO^−^ in bio-imaging and real water samples. Spectrochim. Acta.

