# Supplementary material for: Point-of-Care and Dual-Response Detection of Hydrazine/Hypochlorite-Based on a Smart Hydrogel Sensor and Applications in Information Security and Bioimaging
Source: Molecules. 2023 May 5;28(9):3896. doi: 10.3390/molecules28093896 (PMC10180410; doi:10.3390/molecules28093896)
Supplement: Supplementary file 1 [file molecules-28-03896-s001.zip › molecules-2283745-supplementary.pdf]

Electronic Supplementary Information

Point-of-care and dual-response detection of hydrazine/hypochlorite based on a smart hydrogel sensor and applications in information security and bioimaging

Man Du[a], Yue Zhang[a*], Zhice Xu[a], Zhipeng Dong[b*], Shuchun Zhao[a], Hongxia Du[a], Hua Zhao[a]

[a]College of Chemical and Pharmaceutical Engineering, Hebei University of Science and Technology, Shijiazhuang, 050018, China

[b]Hebei Lansheng Bio-Tech Co., Ltd, Shijiazhuang, 052263, China

Corresponding Author: Yue Zhang, Zhipeng Dong

E-mail addresses: yuezhang@hebust.edu.cn (Yue Zhang);

dzpkjcx@sohu.com (Zhipeng Dong)

Contents:

Table S1 Comparison of fluorescent probes for N_2H_4 and ClO^-. 3

Figure S1 ^1H NMR spectrum of compound **1** in DMSO-d_6. 5

Figure S2 ^{13}C NMR spectrum of probe **1** in DMSO-d_6. 5

Figure S3 HRMS spectrum of compound **1** in CH_3OH. 6

Figure S4 ^1H NMR spectrum of compound **2** in DMSO-d_6. 6

Figure S5 ^{13}C NMR spectrum of probe **2** in DMSO-d_6. 7

Figure S6 HRMS spectrum of compound**2** in CH_3OH. 7

Figure S7 ^1H NMR spectrum of compound **3** in $CDCl_3$-d_1. 8

Figure S8 ^{13}C NMR spectrum of probe **3** in $CDCl_3$-d_1. 8

FigureS9 HRMS spectrum of compound **3** in CH_3OH. 9

Figure S10 ^1H NMR spectrum of probe **XBT-CN** in DMSO-d_6**.** 9

Figure S11 ^{13}C NMR spectrum of probe **XBT-CN** in DMSO-d_6. 10

FigureS12 HRMS spectrum of probe **XBT-CN** in CH_3OH. 10

Figure S13 Effect of pH on the fluorescence intensity of **XBT-CN**. 11

Figure S14 Time dependent fluorescence spectra of **XBT-CN** with added N_2H_4 and ClO^-. 11

Figure S15 HRMS spectrum of **XBT-CN** upon addition of N_2H_4 and ClO^-. 12

DFT Calculations. 14

Kinetic studies. 20

Figure S16-S17 *Pseudo*-first-order kinetic plot. 20

Table S2-S7 Determination of N_2H_4 and ClO^- in real samples. 22

Figure S18 Time-dependent of fluorescence changes of **XBT-CN** in $MPO/H_2O_2/Cl^-$. 25

MTT assay and Bioimaging. 26

Figure S19 Viability of GL261 cells were treated with various concentrations of **XBT-CN**. 26

References 28

Table S1 Comparison of fluorescent probes for N_2H_4 and ClO^-.

Probe structures	$\lambda_{ex}/\lambda_{em}$	Identify objects	Detection limit	Response time	Application
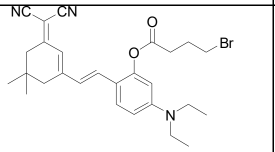 [1]	λ_{ex}=520 nm λ_{em}=680 nm	N_2H_4	1.27 ppb	5 min	Water samples; Living cells; Imaging of zebrafish
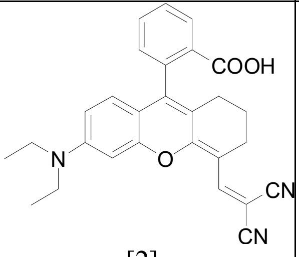 [2]	λ_{ex}=515 nm λ_{em}=565 nm	N_2H_4	0.08 μM	30 min	Water samples; Living cells
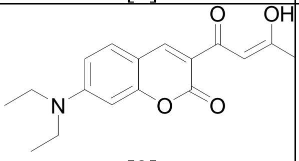 [3]	λ_{ex}=462 nm λ_{em}=516 nm	N_2H_4	1.89 ppb	30 min	Water samples; Living cells
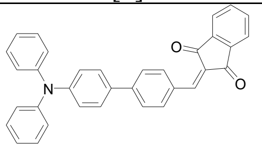 [4]	λ_{ex}=317 nm λ_{em}=470 nm	N_2H_4	1.03 μM	-	Water samples
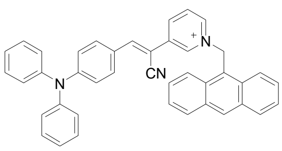 [5]	λ_{ex}=370 nm λ_{em}=414/600nm	ClO^-	65.1 nM	2 min	Living cells
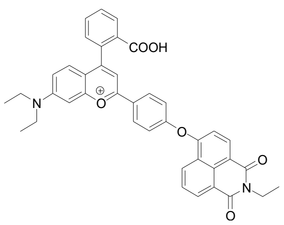 [6]	λ_{ex}=380nm λ_{em}=462/655 nm	ClO^-	25.3 nM	6 min	Living cells
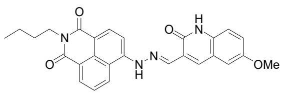 [7]	λ_{ex}=320 nm λ_{em}=479/551 nm	ClO^-	21 nM	20 s	Water samples; Living cells
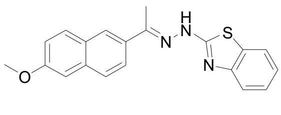 [8]	λ_{em}=445 nm	ClO^-	0.17 μM	-	Tap water; Living cells

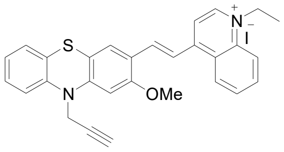 [9]	λ_{ex}=360/460 nm λ_{em}=500/577 nm	N_2H_4 /ClO^-	N_2H_4(89 nM) ClO^- (58 nM)	0.2 min	Living cells
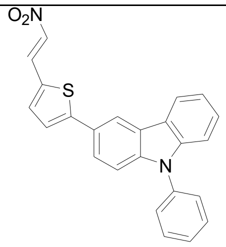 [10]	λ_{em}=434/536 nm	N_2H_4 /ClO^-	N_2H_4(0.6 μM) ClO^-(71.4 nM)	-	Water samples; Living cells
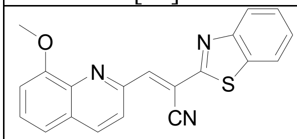 [11]	λ_{ex}=370 nm λ_{em}=455/500 nm	N_2H_4 /ClO^-	N_2H_4(2.25μ M) ClO^-(3.46 μM)	50 s	Living cells
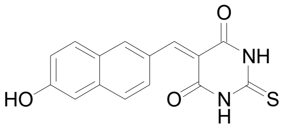 [12]	λ_{ex}=440 nm λ_{em}=482/535 nm	N_2H_4 /ClO^-	N_2H_4(185 nM) ClO^- (64 nM)	30 s	Living cells; Imaging of zebrafish
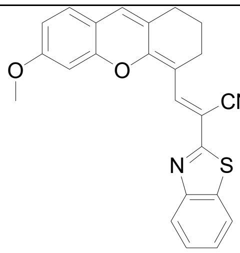 **(This work)**	λ_{ex}=440 nm λ_{em}=470/490 nm	N_2H_4 /ClO^-	N_2H_4(27 nM) ClO^- (34 nM)	20 s	Water samples; Soil samples; Food samples; Encryption ink; Living cells;

"—" Not mentioned.

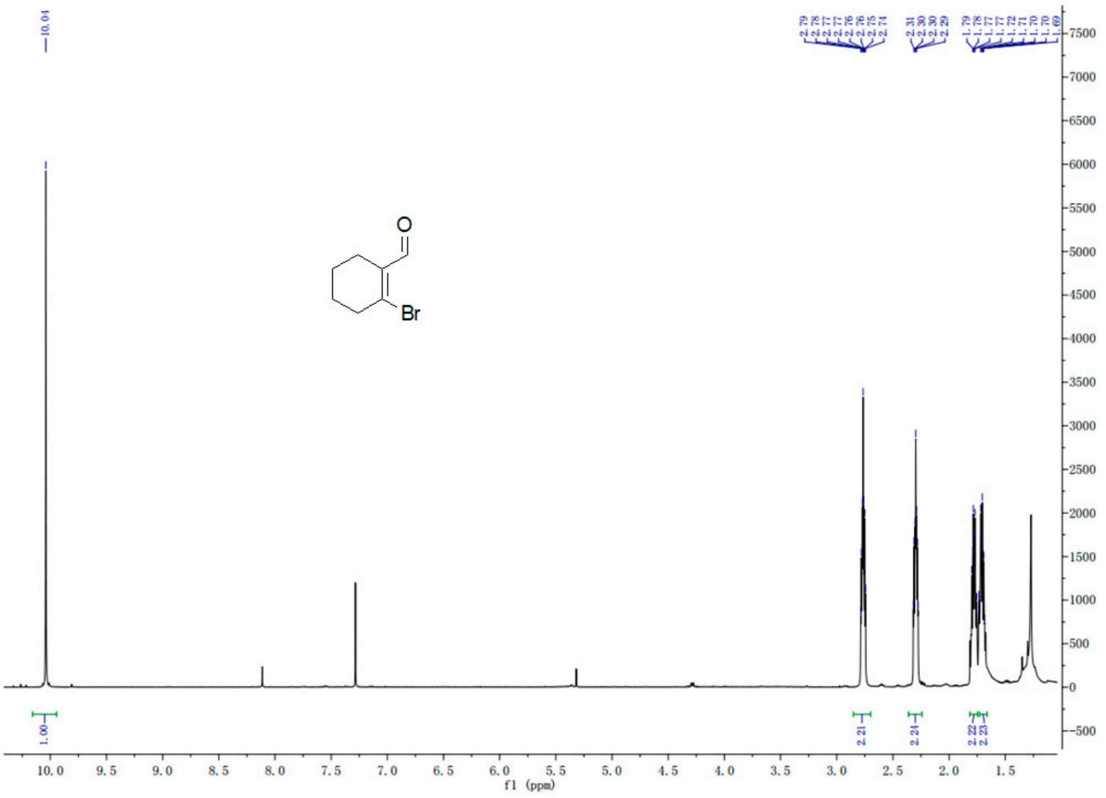

Figure S1. ^{1}H NMR spectrum of compound **1** in DMSO-d_6.

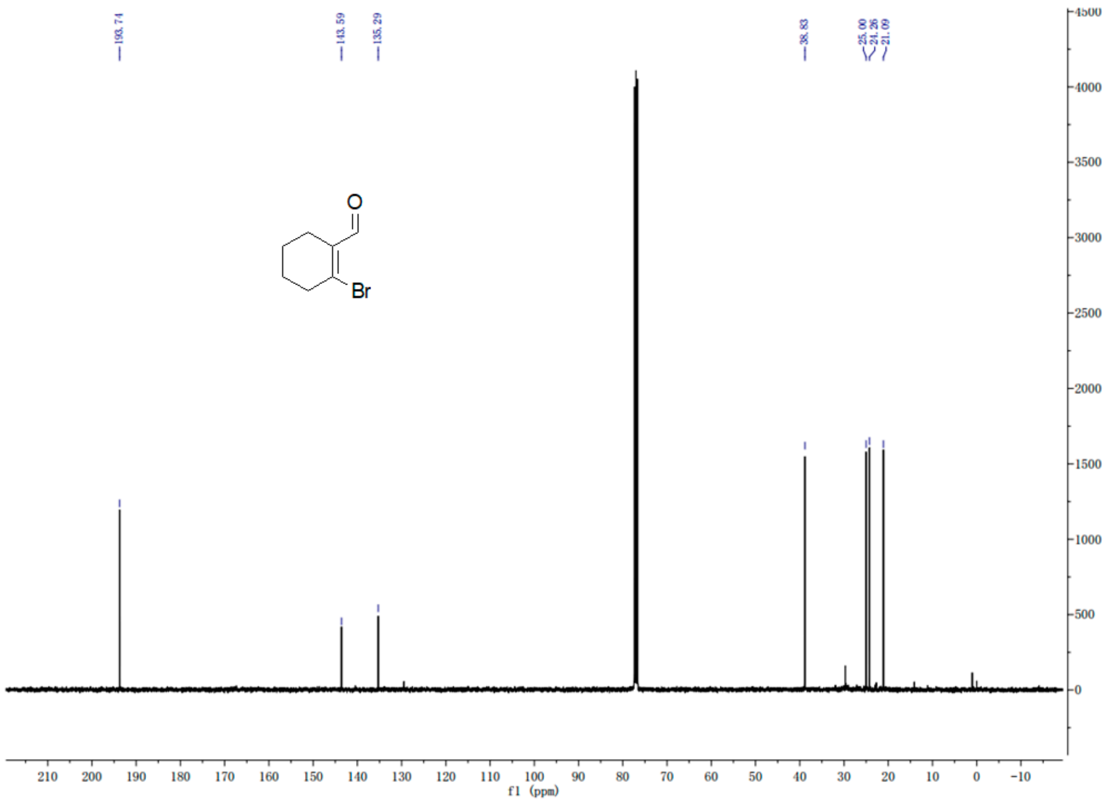

Figure S2. ^{13}C NMR spectrum of compound **1** in DMSO-d_6.

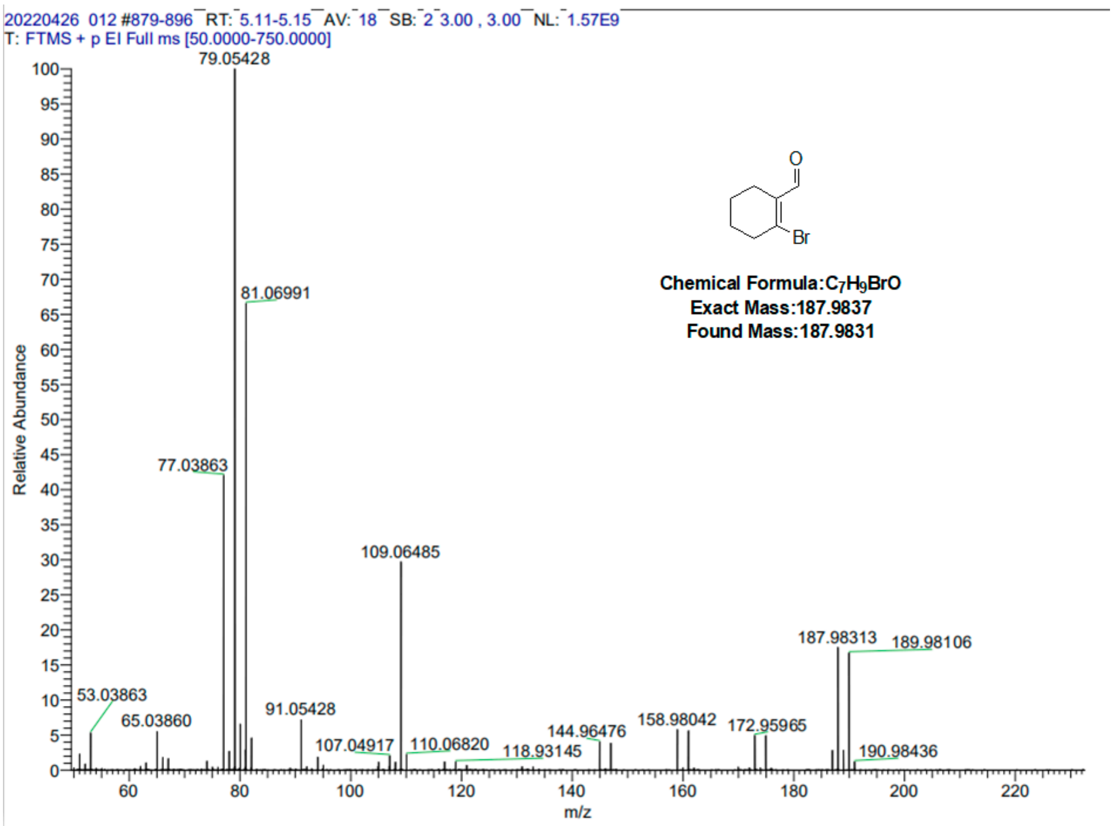

Figure S3. HRMS spectrum of compound **1** in CH_3OH.

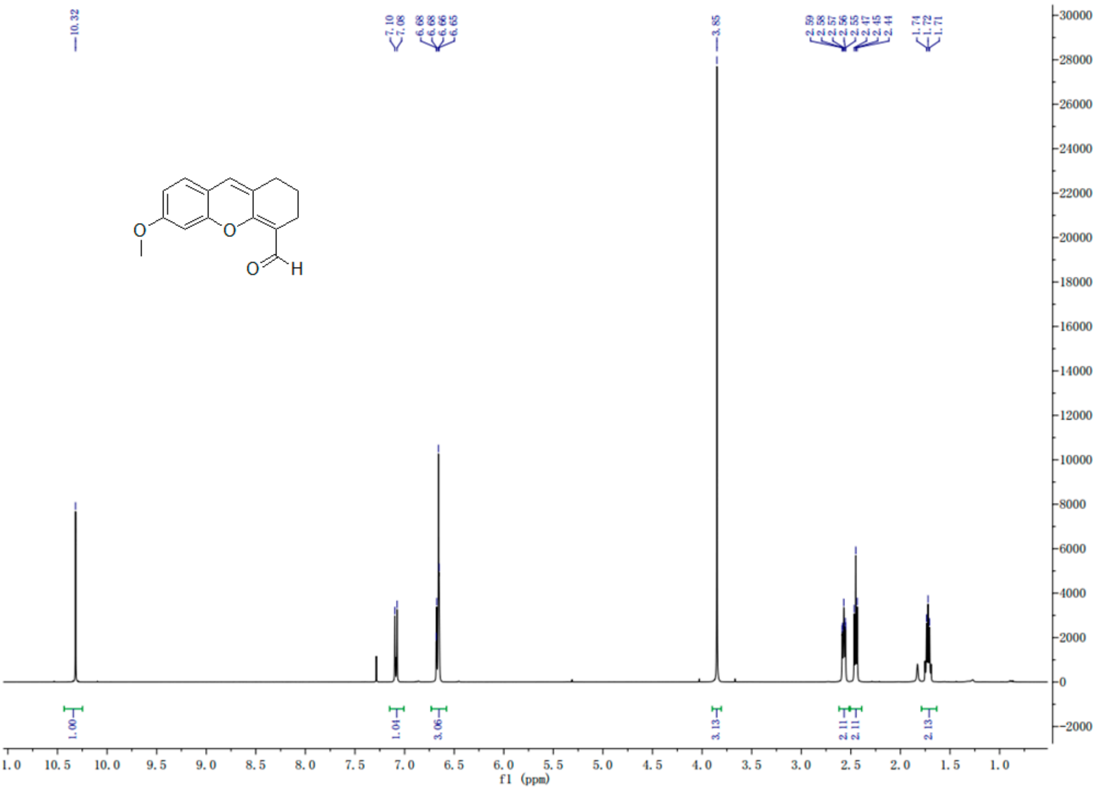

Figure S4. ^{1}H NMR spectrum of compound **2** in DMSO-d_6.

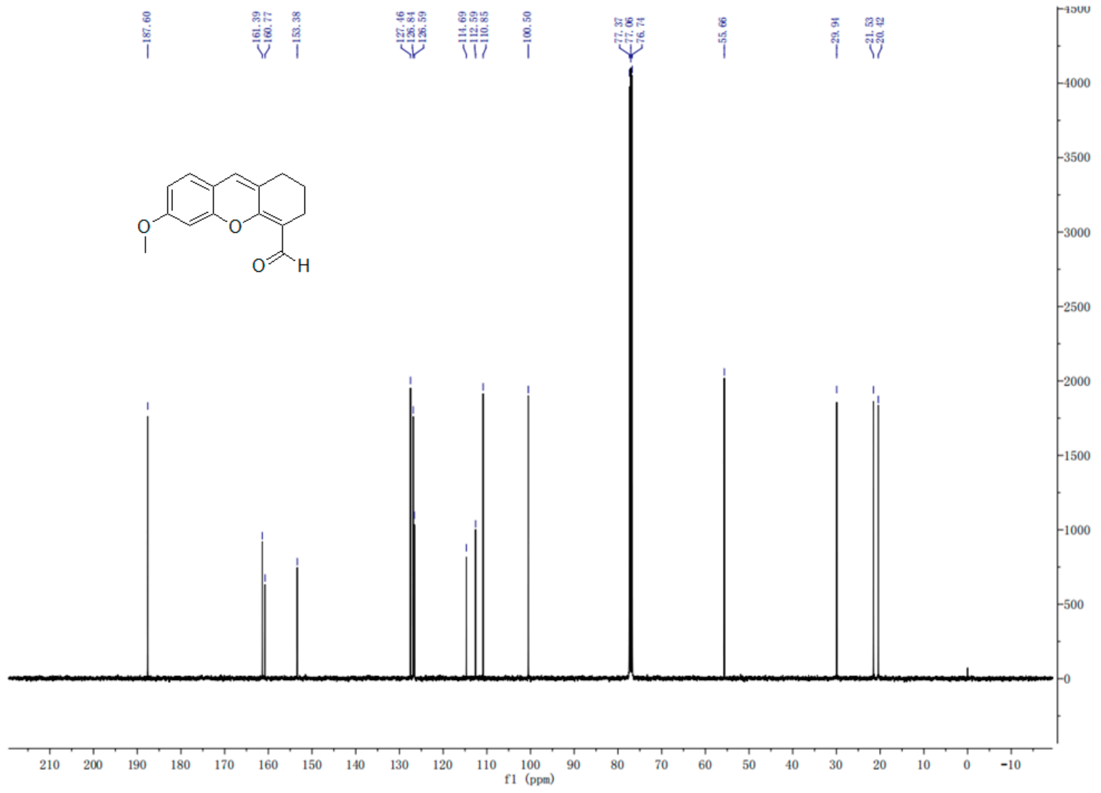

Figure S5. ^{13}C NMR spectrum of compound **2** in DMSO-d_6.

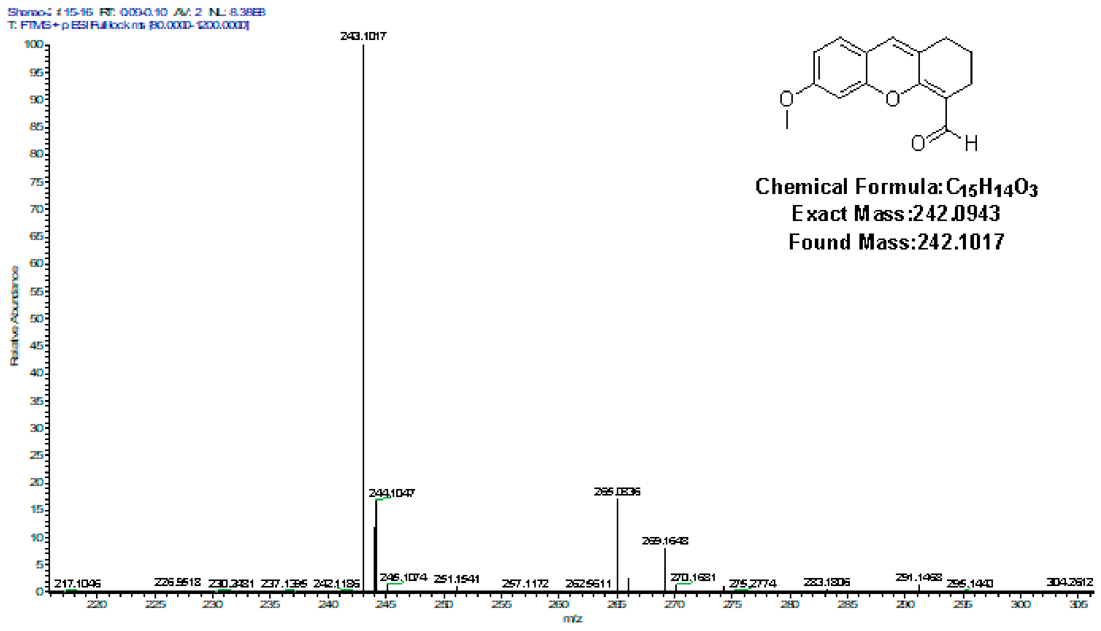

Figure S6. HRMS spectrum of probe compound **2** in CH_3OH.

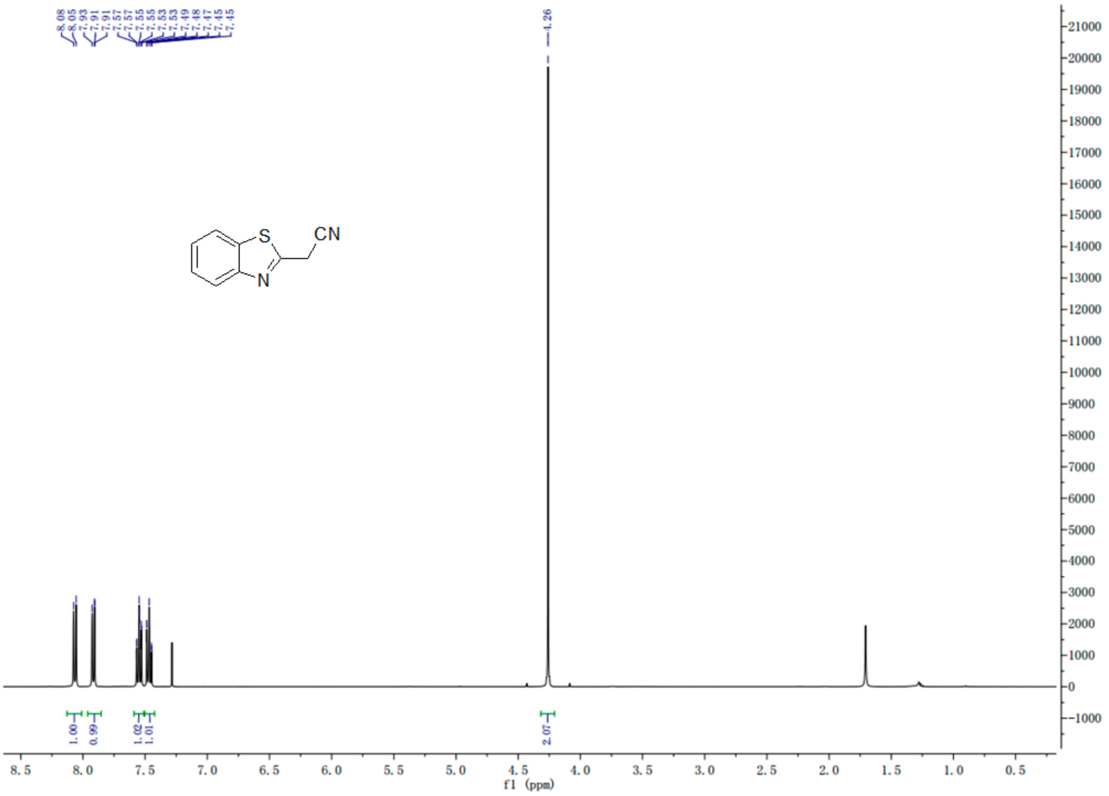

Figure S7. ^{1}H NMR spectrum of compound **3** in $CDCl_3$-d_1.

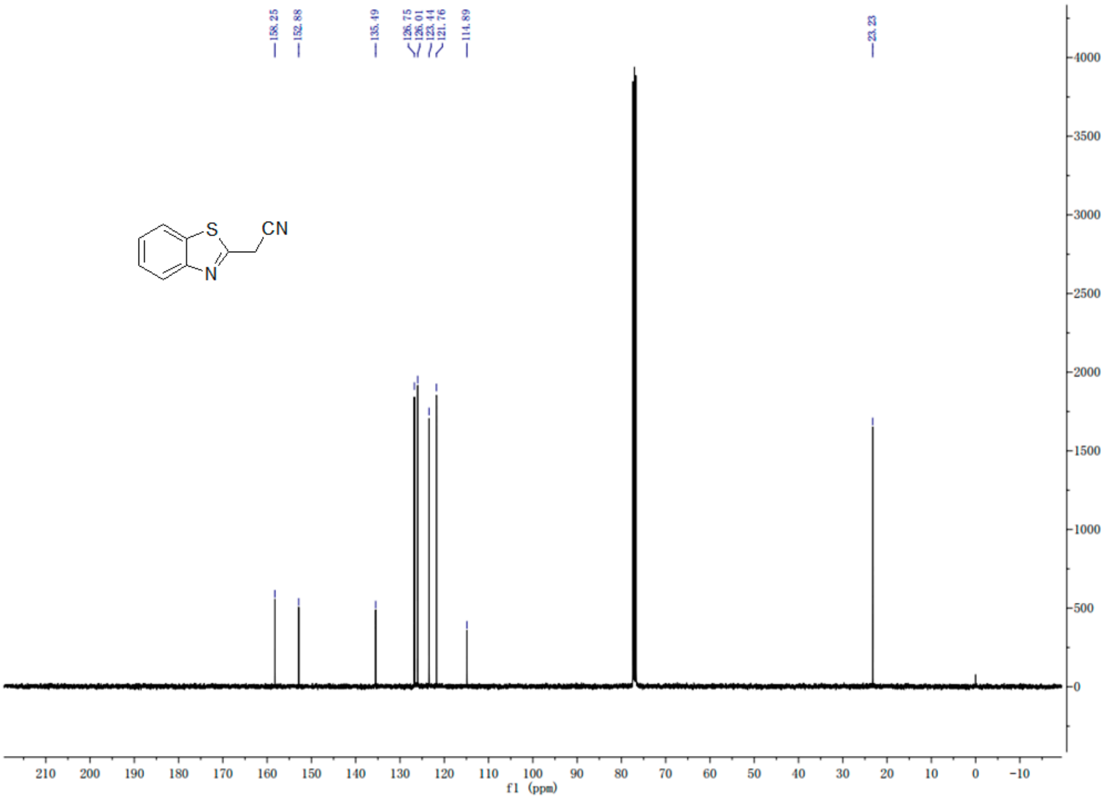

Figure S8. ^{13}C NMR spectrum of compound **3** in $CDCl_3$-d_1.

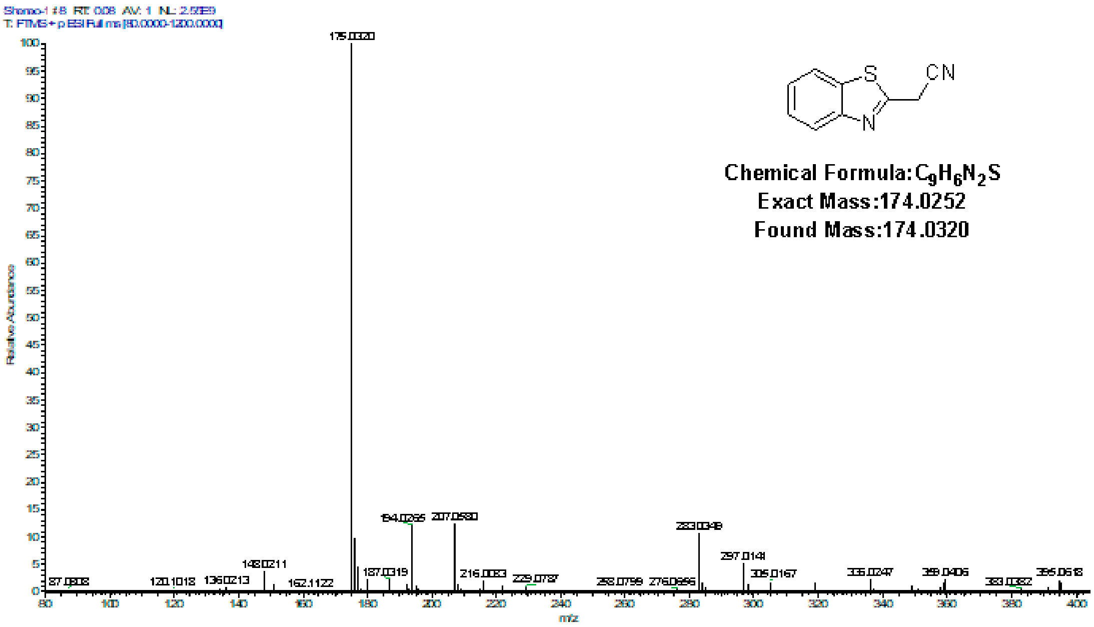

Figure S9. HRMS spectrum of compound **3** in CH_3OH.

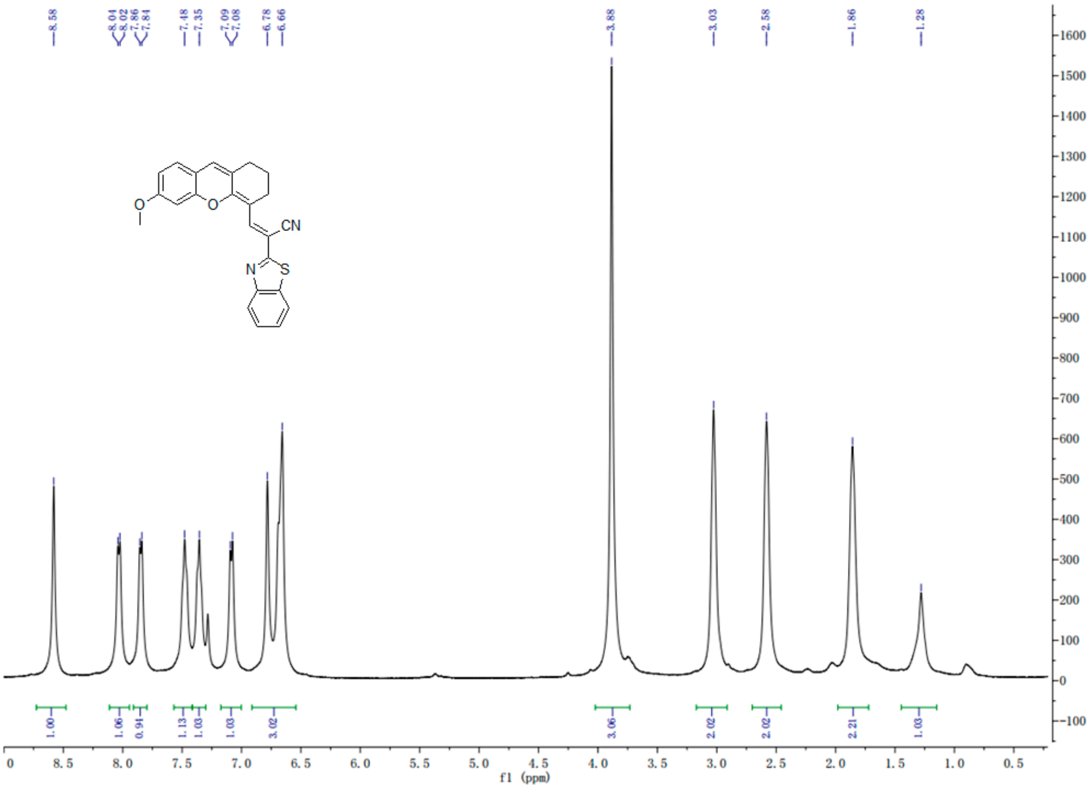

Figure S10. ^1H NMR spectrum of probe **XBT-CN** in DMSO-d_6.

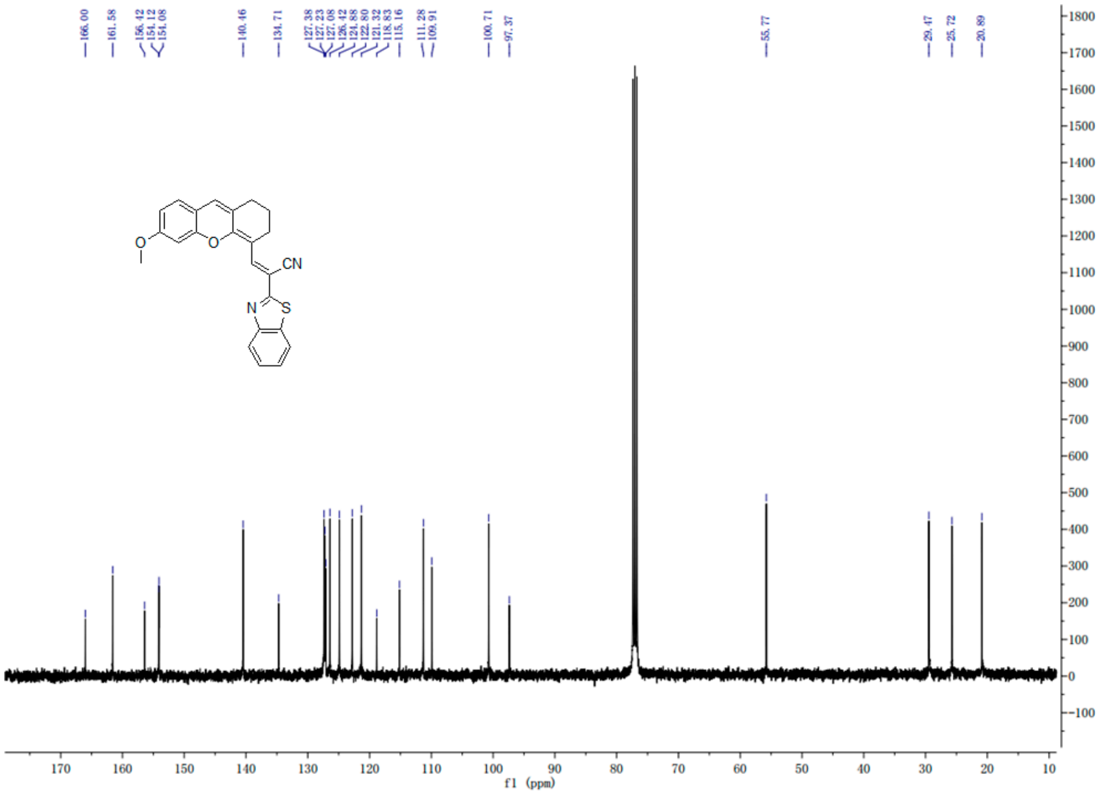

Figure S11. ^{13}C NMR spectrum of probe **XBT-CN** in DMSO-d_6.

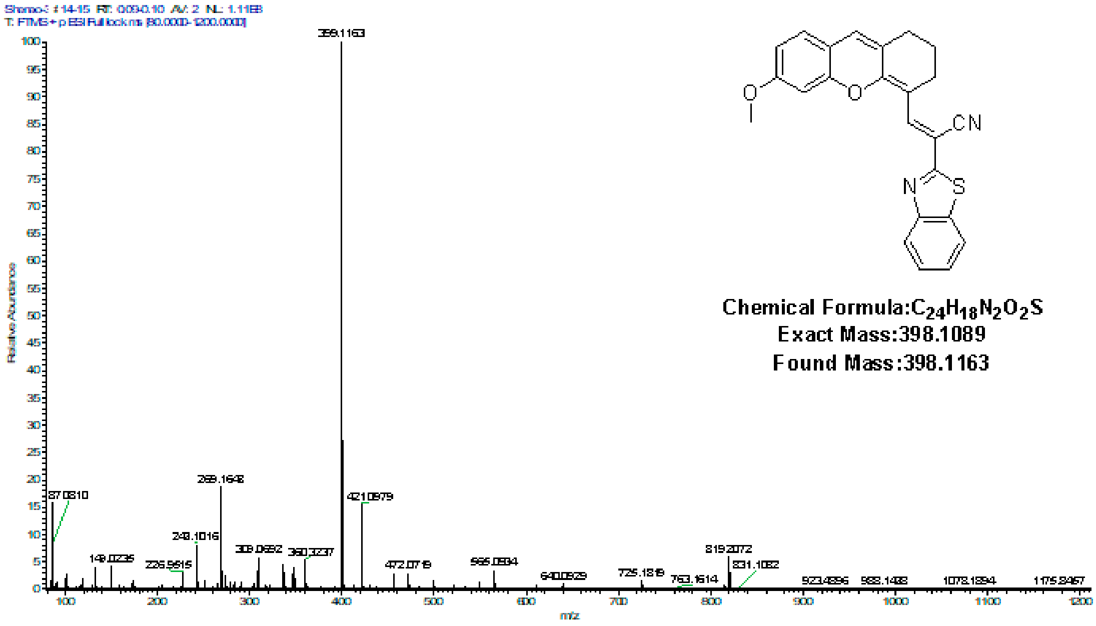

Figure S12. HRMS spectrum of probe **XBT-CN** in CH_3OH.

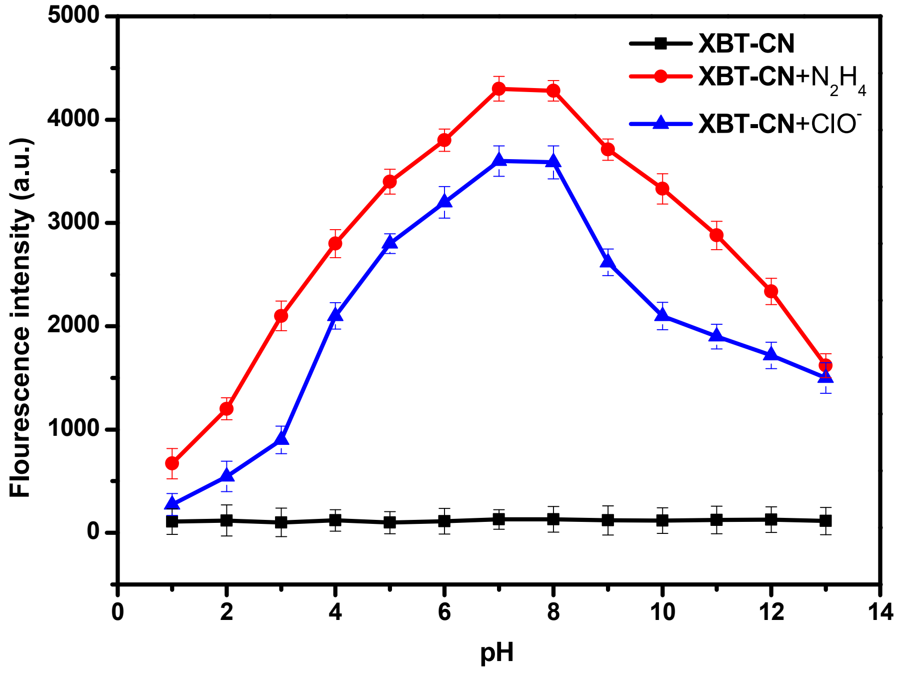

Figure S13. Fluorescence intensity of **XBT-CN** (10 μM) to N_2H_4 (100 μM) and ClO^- (100 μM) in CH_3OH/PBS solution (8/2, v/v) at various pH values.

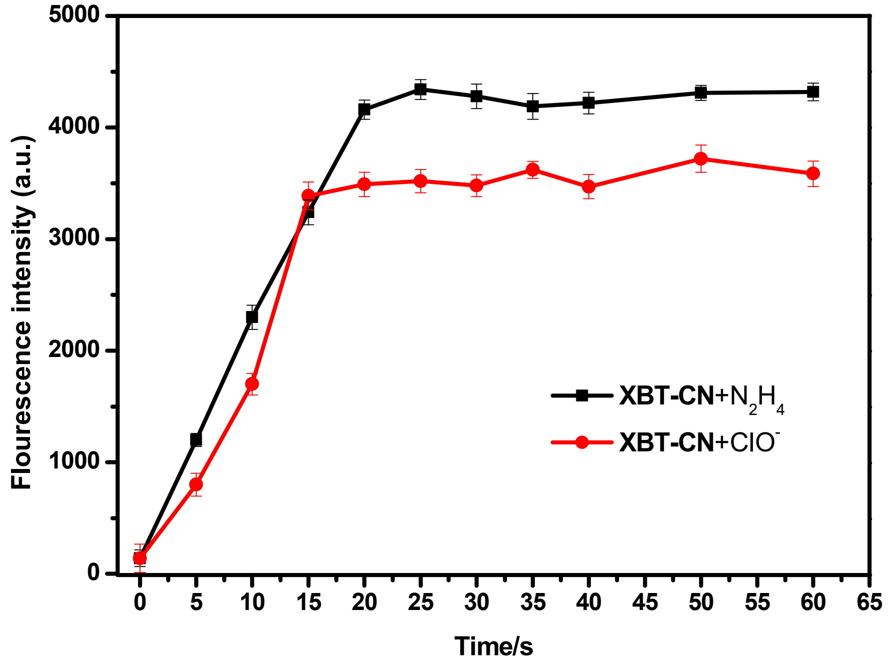

Figure S14. Fluorescence emission spectral changes of **XBT-CN** (10 μM) in CH_3OH/PBS (8/2, v/v) as a function of time upon addition of N_2H_4 (100 μM) and ClO^- (100 μM), respectively.

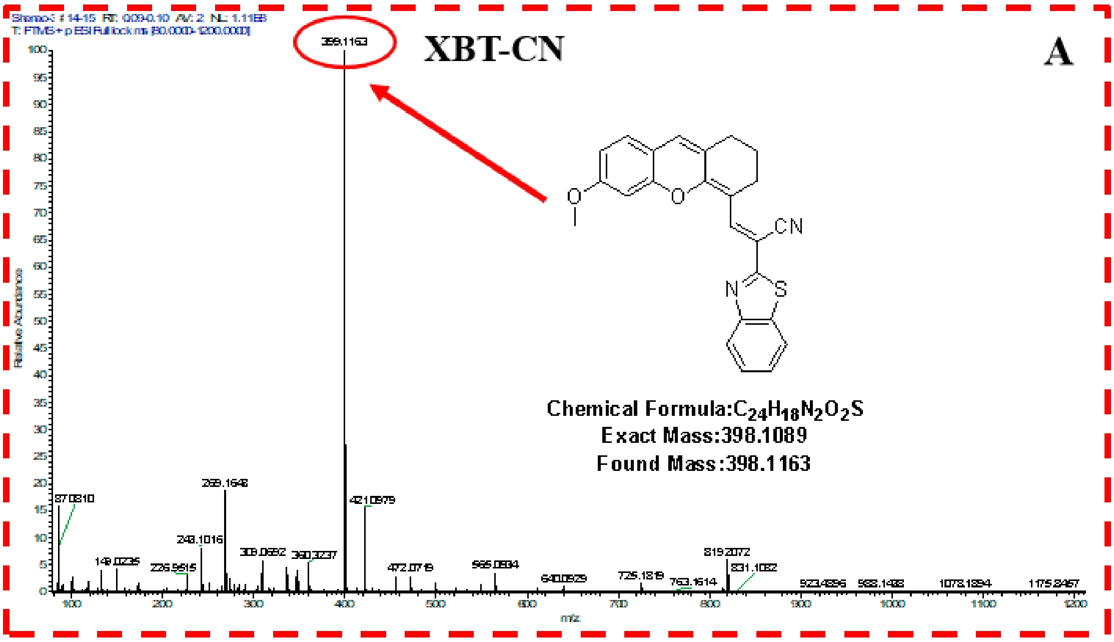
XBT-CN
A
Chemical Formula:$C_{24}H_{18}N_2O_2S$
Exact Mass:398.1089
Found Mass:398.1163

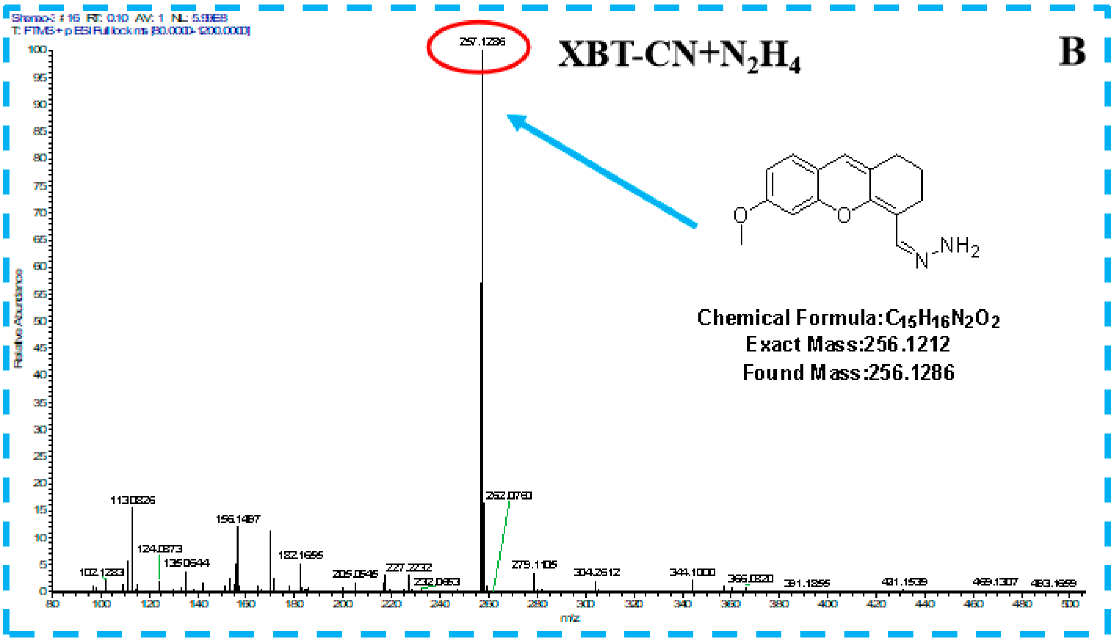
XBT-CN+N_2H_4
B
Chemical Formula:$C_{15}H_{16}N_2O_2$
Exact Mass:256.1212
Found Mass:256.1286

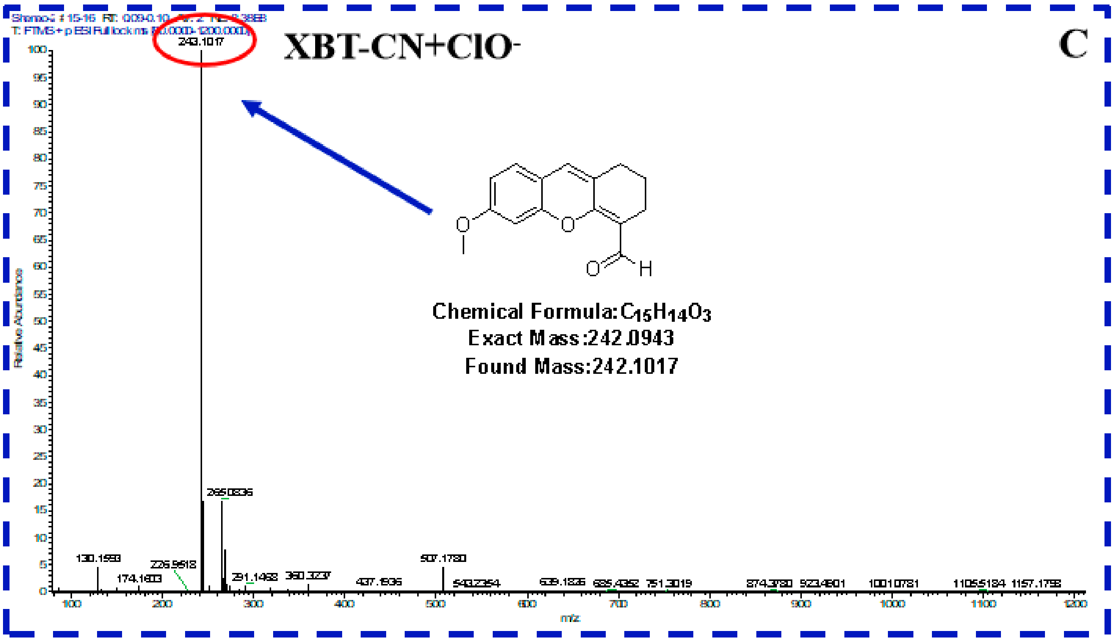

Figure S15. HRMS spectrum of **XBT-CN** (10 μM) (A) in the presence of N_2H_4 (100 μM) (B) and ClO^- (100 μM) (C).

DFT Calculations

Geometric optimizations and energy level calculations were performed in the gas phase at the B3LYP 6-311++G (d, p) level of theory, using the Gaussian 09 software package. Absolute energies in Hartrees are given without additional corrections.

• Cartesian Coordinates for **XBT-CN**

C	4.25147300	2.58694900	0.01650700
C	3.03545500	1.86739400	0.02236500
C	3.06253900	0.47520900	-0.01301400
C	4.28511000	-0.25461100	-0.05865900
C	5.49075100	0.48669300	-0.06328000
C	5.48252100	1.88609400	-0.02581100
C	4.21038100	-1.69742900	-0.10655800
C	3.00385300	-2.36080300	-0.10945100
C	1.76446600	-1.61805400	-0.04054600
C	0.48333900	-2.18460100	-0.01300000
H	2.08990800	2.40198900	0.05627300
H	6.44074300	-0.04949600	-0.09856600
H	6.42650200	2.42632000	-0.03044300
H	5.14163300	-2.26641500	-0.15085800
O	1.83234700	-0.19910400	-0.00045700
O	4.11818000	3.97229900	0.05474900
C	5.35893400	4.78654500	0.05147400
H	5.00213200	5.82330600	0.08369700
H	5.98242900	4.57504100	0.93702100
H	5.94836200	4.61940000	-0.86628600
C	-0.64807500	-1.31574600	-0.01055400
C	-2.01367600	-1.59988200	-0.02306000
H	-0.42854600	-0.24664100	-0.00508600
C	-2.58901600	-2.90314400	-0.05227600

N	-3.10001500	-3.96865900	-0.07758600
C	-2.94172500	-0.47155700	-0.01439700
C	-5.01964200	1.00541800	-0.01085900
C	-3.74138700	1.64981200	0.01059400
C	-6.21033200	1.74089500	-0.00997400
C	-3.68316200	3.06030900	0.03294000
C	-6.12909000	3.14566300	0.01268400
H	-7.17905600	1.24167700	-0.02634700
C	-4.87385100	3.79867500	0.03374600
H	-2.70806800	3.54593700	0.04928000
H	-7.04660500	3.73567700	0.01382500
H	-4.83418100	4.88855600	0.05097300
N	-2.62489700	0.79870300	0.00782700
S	-4.81850800	-0.81038600	-0.03693200
C	2.90408400	-3.88334000	-0.20482000
H	2.80910800	-4.17298300	-1.26918300
H	3.82962400	-4.33675000	0.18774300
C	1.65649100	-4.39420000	0.56751800
H	1.76391000	-4.15085400	1.63828900
H	1.57254700	-5.48880900	0.46803900
C	0.37190100	-3.71874600	0.00838200
H	0.17857400	-4.08936900	-1.01626900
H	-0.49076700	-4.01153200	0.62308100

• Cartesian Coordinates for **XBT-CN-N_2H_4**

C	1.00633300	-1.78158500	0.11168500
C	1.29011800	-0.35918900	0.07435600
C	2.55396600	0.19293500	0.06065100
C	-0.28723000	-2.23566200	0.08083100
C	-1.13493700	0.08336600	0.02552200
C	-1.41224500	-1.31478100	0.03675200
C	-2.76626700	-1.71333700	0.01498700
H	-3.00232300	-2.77933200	0.02386800
C	-3.80891200	-0.77220900	-0.01535600
C	-3.50750200	0.60734200	-0.02294100
C	-2.15852400	1.02976200	-0.00143200
H	-0.49334200	-3.30796000	0.10183700
H	-4.84010400	-1.11836000	-0.03174200
H	-1.92664700	2.09162400	-0.00777800
O	-4.45051100	1.64080000	-0.05163700
O	0.18499600	0.55903400	0.03945600
C	-5.87927500	1.25444200	-0.07184600
H	-6.42096100	2.20873600	-0.09130500
H	-6.12834200	0.66341700	-0.97074700
H	-6.15889400	0.68286600	0.83062800
C	2.74519000	1.62437600	0.05552800
H	1.84015000	2.24756400	0.10534000
N	3.95894300	2.14321500	-0.00404400
N	4.03845100	3.55229200	0.07991400
H	4.90877800	3.90325000	-0.33002900
H	3.18558800	4.09549000	-0.14992000
C	2.21245600	-2.71835000	0.21693100
H	2.47492400	-2.84524200	1.28533500
H	1.94318000	-3.70982000	-0.18487900

C	3.44511100	-2.12653900	-0.52887700
H	3.21208100	-2.04003200	-1.60396800
H	4.31086500	-2.80067200	-0.41531000
C	3.78367400	-0.72166100	0.04372100
H	4.17847500	-0.82602700	1.07296600
H	4.56720000	-0.22619600	-0.55101800

• Cartesian Coordinates for **XBT--CN-ClO$^-$**

C	1.45316400	-1.35671400	0.11383000
C	1.53662000	0.09718200	0.08997400
C	2.72039600	0.80092500	0.07999400
C	0.23072700	-1.97706500	0.07636400
C	-0.92149800	0.20109100	0.03319200
C	-1.00565400	-1.22034700	0.03839300
C	-2.29452100	-1.80100800	0.01154700
H	-2.38377400	-2.88898800	0.01538500
C	-3.44999000	-1.00908400	-0.01788900
C	-3.33856500	0.40230700	-0.02036900
C	-2.06043900	1.00396300	0.00576400
H	0.17080600	-3.06791600	0.08124900
H	-4.42605200	-1.48848700	-0.03827700
H	-1.97236100	2.08713700	0.00219600
O	-4.41068600	1.29324600	-0.04758400
O	0.32518700	0.84611000	0.05312800
C	-5.77899600	0.72249100	-0.07831000
H	-6.43824400	1.59924200	-0.09523900
H	-5.94266400	0.11159100	-0.98288900
H	-5.98441900	0.11501100	0.82002500
C	2.77486700	-2.12572800	0.19663900
H	3.05410500	-2.23992800	1.26210100
H	2.63768900	-3.13523700	-0.22621300
C	3.92006500	-1.35998300	-0.53424400
H	3.68101400	-1.28434200	-1.60873400
H	4.86622800	-1.91615200	-0.42755900
C	4.05772000	0.06292800	0.07081900
H	4.44593700	-0.01406200	1.10542300
H	4.77008400	0.68896800	-0.49094900

C	2.76941600	2.26128200	0.04173400
O	3.84386200	2.91860400	-0.02144200
H	1.77488300	2.75604600	0.06603400

Kinetic studies:

The reaction of **XBT-CN** (10 μM) with N_2H_4/ClO^- in CH_3OH/PBS solution (8/2, v/v, pH 7.4) was monitored using the fluorescence intensity at 470/490 nm. The reaction was carried out at 25 °C. The *pseudo*-first-order rate constant for the reaction was determined by fitting the fluorescence intensities of the samples to the *pseudo*-first-order equation:

$$\mathrm{Ln}\ [(F_{max} - F_t) / F_{max}] = - k't$$

Where F_{max} and F_t are the fluorescence intensities at 470/490 nm at time t and the maximum value obtained after the reaction was complete. *k'* is the *pseudo*-first-order rate constant. The *pseudo*-first-order plots for the reaction of **XBT-CN** with 10 equiv. of N_2H_4/ClO^- is shown in Figure S16 and Figure S17, the *pseudo*-first-order rate constant $k' = 1/t_1 = 0.3656\ \mathrm{min^{-1}}$ and $0.3374\ \mathrm{min^{-1}}$ for N_2H_4 and ClO^- respectively.

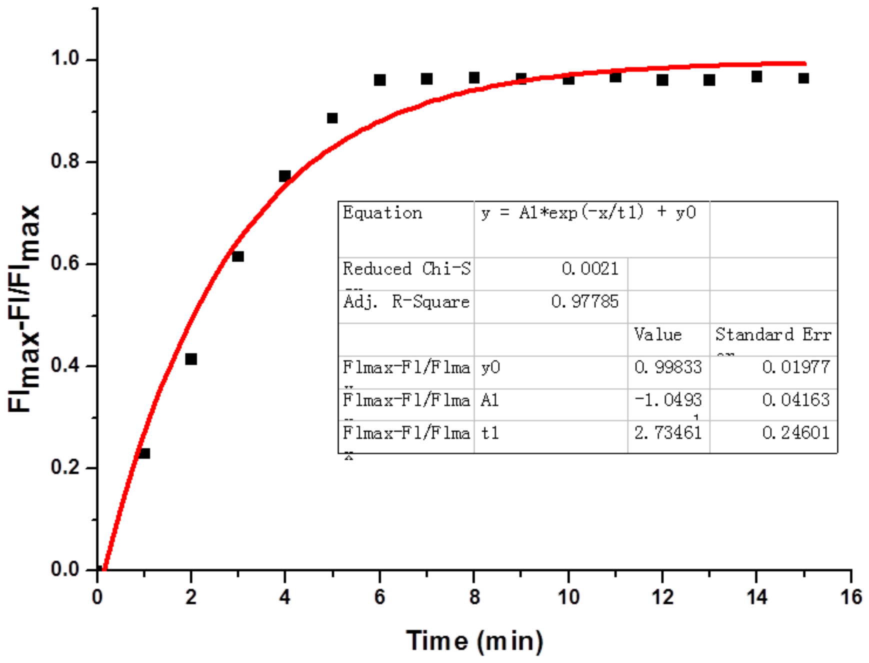

Figure S16. *Pseudo*-first-order kinetic plot of the reaction of **XBT-CN** (10 μM) with N_2H_4 (10 equiv.) in CH_3OH/PBS solution (8/2, v/v, pH 7.4), $k = 0.3656\ \mathrm{min^{-1}}$.

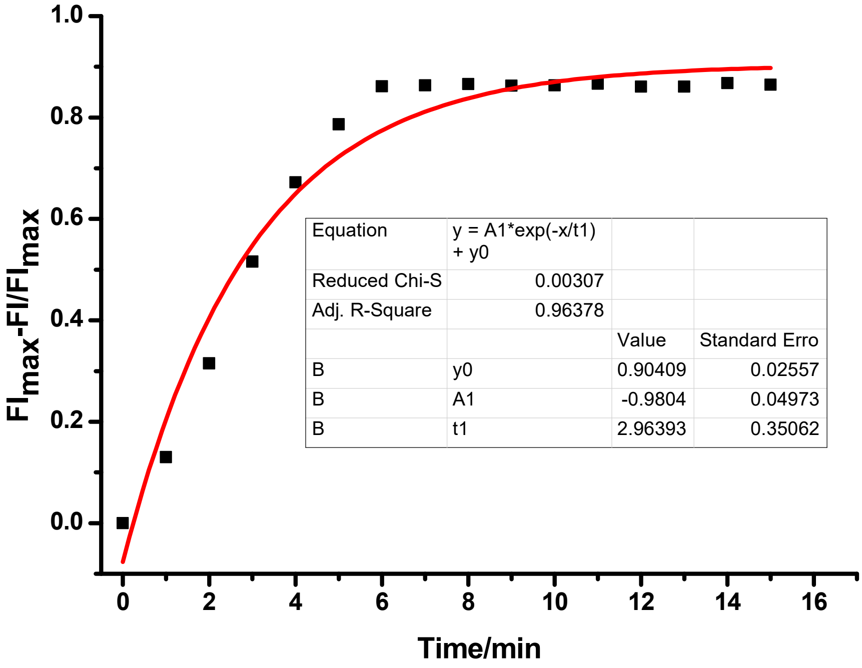

Figure S17. *Pseudo*-first-order kinetic plot of the reaction of **XBT-CN** (10 μM) with ClO^- (10 equiv.) in CH_3OH/PBS solution (8/2, v/v, pH 7.4), k =0.3374 min^{-1}.

Table S2. Validation of N_2H_4 detection method in real water samples.

Samples	Standard	Spectroscopic method		Hydrogel test kit	
	$[N_2H_4]$/ μM	$[N_2H_4]$/ μM	Recovery rate(%)	$[N_2H_4]$/ μM	Recovery rate(%)
Tap water	0	-	-	-	-
	5.00	5.00	103	5.00	104
	10.00	10.00	105	10.00	103
Lake water	0	-	-	-	-
	5.00	5.00	100	5.00	101
	10.00	10.00	102	10.00	98
River Water	0	-	-	-	-
	5.00	5.00	97	5.00	103
	10.00	10.00	101	10.00	105
Industrial Wastewater	0	-	-	-	-
	5.00	5.00	98	5.00	102
	10.00	10.00	100	10.00	104

Table S3. Validation of ClO^- detection method in real water samples.

Samples	Standard	Spectroscopic method		Hydrogel test kit	
	$[ClO^-]$/ μM	$[ClO^-]$/ μM	Recovery rate(%)	$[ClO^-]$/ μM	Recovery rate(%)
Tap water	0	-	-	-	-
	5.00	5.00	102	5.00	103
	10.00	10.00	104	10.00	99
Lake water	0	-	-	-	-
	5.00	5.00	99	5.00	106
	10.00	10.00	101	10.00	104
Mineral Water	0	-	-	-	-
	5.00	5.00	98	5.00	105
	10.00	10.00	99	10.00	101
Industrial Wastewater	0	-	-	-	-
	5.00	5.00	97	5.00	102
	10.00	10.00	103	10.00	104

Table S4. Validation of N_2H_4 detection method in soil samples.

Samples	Standard	Spectroscopic method		Hydrogel test kit	
	$[N_2H_4]/\mu M$	$[N_2H_4]/\mu M$	Recovery rate(%)	$[N_2H_4]/\mu M$	Recovery rate(%)
Clay soil	0	-	-	-	-
	5.00	5.00	104	5.00	103
	10.00	10.00	105	10.00	98
Field soil	0	-	-	-	-
	5.00	5.00	100	5.00	99
	10.00	10.00	102	10.00	103
Sand soil	0	-	-	-	-
	5.00	5.00	99	5.00	101
	10.00	10.00	103	10.00	104

Table S5. Validation of ClO^- detection method in soil samples.

Samples	Standard	Spectroscopic method		Hydrogel test kit	
	$[ClO^-]/\mu M$	$[ClO^-]/\mu M$	Recovery rate(%)	$[ClO^-]/\mu M$	Recovery rate(%)
Clay soil	0	-	-	-	-
	5.00	5.00	102	5.00	101
	10.00	10.00	103	10.00	105
Field soil	0	-	-	-	-
	5.00	5.00	104	5.00	103
	10.00	10.00	101	10.00	104
Sand soil	0	-	-	-	-
	5.00	5.00	99	5.00	101
	10.00	10.00	97	10.00	98

Table S6. Validation of N_2H_4 detection method in food samples.

Samples	Standard	Spectroscopic method		Hydrogel test kit	
	$[N_2H_4]/\mu M$	$[N_2H_4]/\mu M$	Recovery rate(%)	$[N_2H_4]/\mu M$	Recovery rate(%)
Rice	0	-	-	-	-
	5.00	5.00	104	5.00	100
	10.00	10.00	103	10.00	98
Flour	0	-	-	-	-
	5.00	5.00	102	5.00	103
	10.00	10.00	104	10.00	104
Beer	0	-	-	-	-
	5.00	5.00	101	5.00	99
	10.00	10.00	105	10.00	102
Cabbage	0	-	-	-	-
	5.00	5.00	97	5.00	103
	10.00	10.00	99	10.00	101

Table S7. Validation of ClO^- detection method in food samples.

Samples	Standard	Spectroscopic method		Hydrogel test kit	
	$[ClO^-]/\mu M$	$[ClO^-]/\mu M$	Recovery rate(%)	$[ClO^-]/\mu M$	Recovery rate(%)
Rice	0	-	-	-	-
	5.00	5.00	103	5.00	98
	10.00	10.00	100	10.00	102
Flour	0	-	-	-	-
	5.00	5.00	102	5.00	103
	10.00	10.00	104	10.00	100
Beer	0	-	-	-	-
	5.00	5.00	106	5.00	103
	10.00	10.00	101	10.00	104
Cabbage	0	-	-	-	-
	5.00	5.00	98	5.00	102
	10.00	10.00	99	10.00	100

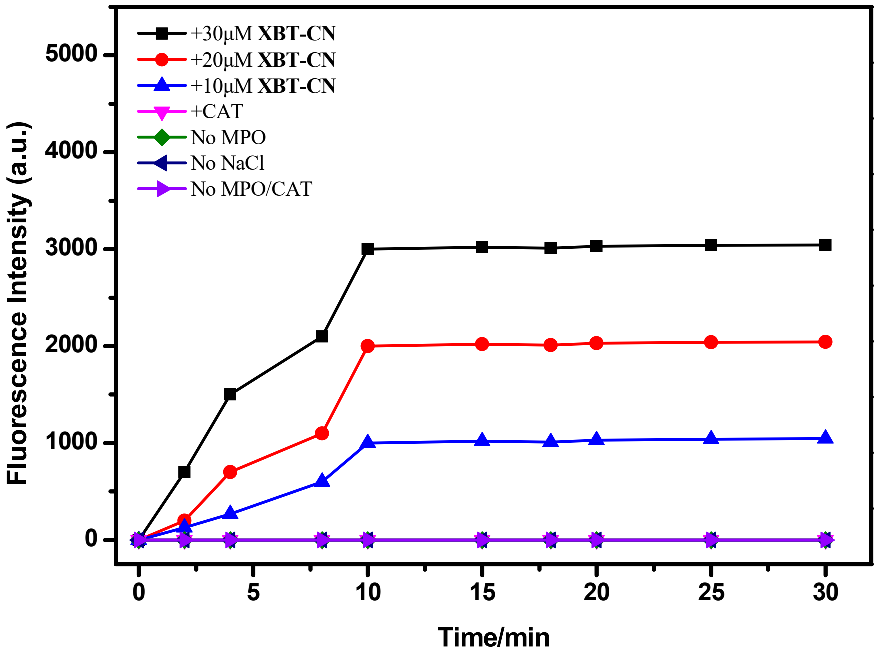

Figure S18. Time-dependent of fluorescence changes of the incubation mixture containing **XBT-CN** (5 μM) and MPO/H_2O_2/Cl^- at pH 7.4 (0.034 U/ml MPO, 10 μM H_2O_2, 0.1 M NaCl, 50 mM phosphate buffer).

MTT assay and Bioimaging

1. Cell culture

GL261 cells (mouse glioma cells) were cultured in Dulbecco's modified medium (DMEM) supplemented with 10% fetal bovine serum (FBS) and 1% antibiotics penicillin (PS). The medium is replaced at regular intervals (24 to 48 h) and the cells was incubated at 37°C under an atmosphere of 5% CO_2.

2. Cell viability assay

GL261 cells were seeded in 96-well plates and incubated for 24 h at 37°C and 5% CO_2. After that the cells were removed the medium and washed with PBS (3 times), and then incubated with fresh medium at various concentrations of **XBT-CN** (0, 10, 20, 30, 40, and 50 μM). After 48 h incubation, the cells were washed PBS and treated with the DMEM containing MTT (0.5 mg/mL). This MTT assays were performed using a Thermo MK3 ELISA plate reader. The independent experiments performed in six replicates were used to obtain the statistical mean and standard deviation.

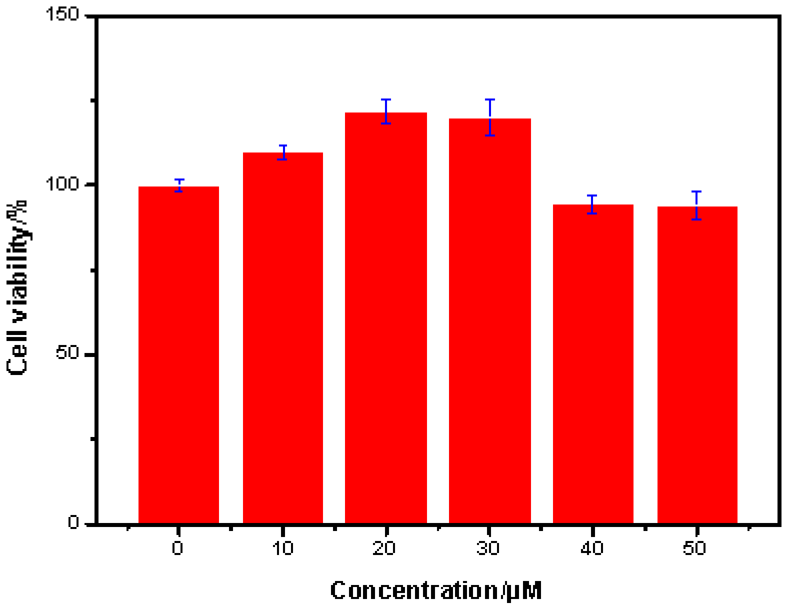

Figure S19. Viability of GL261 cells were treated with various concentrations of probe **XBT-CN** (0, 10, 20, 30, 40, and 50 μM) for 48 h.

3. Cell imaging

The cells were seeded in 6-well plates and incubated for 24 h until the confluence

50%-70%, and then washed with the PBS (3 times), and then incubated with the DMEM medium containing the **XBT-CN** (20 μM) at 37ºC and 5% CO_2. After 2 h incubation, the cells were washed with PBS and incubated with the DMEM medium containing ClO^- (80 μM) or N_2H_4 (100 μM) for 2 h before imaging. The fluorescence images were obtained using an Olympus IX71 inverted fluorescence microscope.

References

[1] M. Q. Zhu, Z. Y. Zhao, Y. Huang, F. G. Fan, F. Wang, W. L. Li, X. W. Wu, R. M. Hua and Y. Wang, *Sci. Total Environ.*, 2021, **759**, 143102.

[2] S. Mu, H. Gao, C. Li, S. S. Li, Y. Y. Wang, Y. Zhang, C. M. Ma, H. X. Zhang and X. Y. Liu, *Talanta*, 2021, **221**, 121606.

[3] R. Chen, G. J. Shi, J. J. Wang, H. F. Qin, Q. Zhang, S. J. Chen, Y. H. Wen, J. B. Guo, K. P. Wang and Z. Q. Hu, *Spectrochim Acta*, 2021, **252**, 119510.

[4] D. H. Li, L. Liu, H. G. Yang, J. Ma , H. L. Wang and J. M. Pan, *Materials Science and Engineering B*, 2022, **276**, 115556.

[5] X. H. Huang, T. C. Luo, C. Zhang, J. R. Li, Z. J. Jia, X. L. Chen, Y. J. Hu and H. Huang, *Talanta*, 2022, **241**, 123235.

[6] X. H. Xu, H. C. Ding, Q. Zhang, G. Liu and S. Z. Pu, *Dyes and Pigments*, 2022, **207**, 110776.

[7] C. L. Zhang, X. L. Li, Y. H. Jiang, Y. N. Zhang, Y. X. Xie, Y. D. Sun and C. Liu, *Spectrochimic Acta*, 2022, **283**, 121736.

[8] N. N. Li, Y. E. Gao, X. Y. Xu, P. Qiu, Y. Gao, M. Yan, Q. Zhang, W. Y. Lin, Z. Y. Xing and Z. A. Zong, *Dyes and Pigments*, 2023, **210**, 110965.

[9] B. T. Zhu, X. L. Wu, J. Rodrigues, X. C. Hu, R. L. Sheng and G. M. Bao, *Spectrochim. Acta*, 2021, **246**, 118953.

[10] L. Wang, Q. Pan, Y. Chen, Y. F. Ou, H. Y. Li and B. W. Li, *Spectrochim. Acta*, 2020, **241**, 118672.

[11] S. Das, L. Patra, P. P. Das, K. Ghoshal, S. Gharami, J. W. Walton, M. Bhattacharyyae and T. K. Mondal, *Phys. Chem. Chem. Phys.*, 2022, **24**, 20941-20952.

[12] X. J. He, Z. A. Deng, W. Xu, Y. H. Li, C. C. Xu, H. Chen and J. L. Shen, *Sens. Actuators B*, 2020, **321**, 128450.
